# Trademark potential increase and entrepreneurship rural development: A case study of Southern Transylvania, Romania

**DOI:** 10.1371/journal.pone.0245044

**Published:** 2021-01-15

**Authors:** Daniel Stefan, Valentina Vasile, Maria-Alexandra Popa, Anca Cristea, Elena Bunduchi, Cezar Sigmirean, Anamari-Beatrice Stefan, Calin-Adrian Comes, Liviu Ciucan-Rusu

**Affiliations:** 1 Center for Law, Economics and Business Studies, "George Emil Palade" University of Medicine, Pharmacy, Science and Technology of Târgu-Mureș, Târgu-Mureș, MUREȘ County, Romania; 2 Institute of National Economy, Romanian Academy, Bucharest, Romania; 3 Department of Sciences and Letters, "George Emil Palade" University of Medicine, Pharmacy, Science and Technology of Târgu-Mureș, Târgu-Mureș, MUREȘ County, Romania; The Bucharest University of Economic Studies, ROMANIA

## Abstract

Cultural heritage capitalization in rural areas redefines the local development model. Thus, heritage tourism has become the engine of economic activities diversification. This study aims to identify a decision-making-model substantiating algorithm in order to support the local heritage capitalization (lesser known on the international cultural consumption market), based on three types of qualitative researches, and the improved Analytical Hierarchy Process (AHP) method. In case of lesser known heritage, trademark potential and international cultural tourism route for heritage capitalization are smart choices for the innovative local hub development. The developed AHP version allows for a broader investigation of the characteristics that can lead to a trademark associated development based on integrated and innovative tourism products. To substantiate our approach and validate the model, we conducted a pilot study on a geographic area (Southern Transylvania, Romania), slightly exploited from the perspective of heritage potential, and characterized by a combination of heritage assets. The study’s results can be used by local authorities as a foundation for sound and strategic development of the area with economic potential from tangible and intangible heritage (re)interpretation.

## 1. Introduction

Heritage has a complex meaning, which includes tangible and intangible elements defining time and space that can be associated with culture and nature. Through tourism, cultural heritage can be transformed into an important resource for local economic development [1]. With the growing demand for experiential cultural tourism (in which tourists can experience country-side life, can learn local traditions, crafts, etc.), rural tourism is tackling bold innovative ideas to stimulate cultural consumption [2]. However, in a market economy, rapid changes caused by technology, economic crises, or other macroeconomic phenomena can hamper the economic agents’ efforts to meet consumer demand. Therefore, the European Union has developed a strategy to support the local economy through smart specialization, which aims to capitalize on existing strengths in a local economy, highlight hidden opportunities, and create useful platforms for local / regional development through smart choices [3].

A trademark is used to distinguish one entity’s goods or services [4, 5]. It is increasingly becoming important for businesses in the digital era, with brand and reputation as valuable assets for increasing attractivity among cultural consumers and tourists. In cultural industries, the trademark does not necessarily refer to patents, but rather to "soft innovation" which includes intellectual forms of innovation [6]. World Intellectual Property Organization (WIPO) identified the term”Traditional Cultural Expressions” (TCEs), which include forms of traditional culture expressions (such as handicrafts, art, music, dance or other cultural expressions) that form part of the identity and heritage of a community that are transmitted from generation to generation [7]. These forms of cultural expressions may be protected by intellectual property rights, such as copyrights or trademarks. The link between cultural heritage and intellectual property has been studied by the WIPO for many decades [8]. In case of lesser known heritage, trademark potential and cultural tourism for capitalization enhance the innovative rural hub development [9, 10].

Cultural tourism development is conditioned by regional strategic planning and resource allocation from public bodies and the entrepreneurs’ level of involvement [11], which must provide products and services adapted to the tourists’ needs, depending on the opportunities offered by the area. Moreover, the digital economy stimulates innovative cultural entrepreneurship hence supporting the development of small businesses by creating collaborative networks for integrated tourism, combining various forms of cultural tourism consumption—from a) accommodation services in traditional houses and culinary experiences that enhance the local cuisine, to b) participatory forms of acquiring knowledge on the traditions and customs promoting the local history of the place through social networks and c) in-situ experiences–life in the villages, learning habits of work and the day to day life in rural areas, making traditional objects/handicrafts, etc.

Digitalization and social media have promoted higher basic cultural heritage knowledge. It is proven that the presence in social media of heritage tourism positively affects tourism entrepreneurship [12]. Therefore, any new heritage asset capitalized for cultural offer is a smart choice for boosting local business development. In this context, trademark becomes a driving force for joint cultural heritage (built heritage assets and local intangible assets) and can support innovative business models and hub development.

Tourism has been recognized for stimulating economic development, increasing exchange, smallholder investment, and local employment [13]. However, studies on cultural heritage capitalization in rural areas are few [14–17]. Previous research pointed out the failure of regional planning to include new changes and developments in rural area [18]. However, some rural tourism related academic developments could be considered contributory to spatial development boundaries, opportunities, visual impact, and the overall environment [19]. Some also contribute to the planning processes by using a multi-criteria decision making method, namely the Analytical Hierarchy Process (AHP), to identify a suitable location for the development of different tourism assets, in order to fit into the environment [20].

In the past decades, Southern Transylvania–Romania, has developed an increased interest of various categories of niche tourism consumers–who wish to experience daily life "returning to the wild natural ecosystems" and authentic cultural consumption, based on local heritage. Transylvania is recognized for several world heritage like Sighisoara town—a small scale fortified living citadel built in the 12th century [21], Alba Iulia—the largest Vauban fortress in South East Europe [22] or the seven Saxons villages UNESCO listed. Even more, Southern Transylvania hides small villages almost unknown even by the Romanians with unrevealed tourist heritage assets. Such an authentic mix of heritage components and a natural landscape minimally affected by progress marks Transylvania as an attractive pole of interest for cultural consumers. Old built heritage restoration, the revival of local traditions and customs and the development of cultural consumption based on combining digitalization with in-situ experiences define the local development’s value chain. Therefore, we considered Southern Transylvania a suitable example for our investigation which aims to understand the importance of cultural heritage capitalization for local economic development in an internationally recognized mythical multicultural area. In our research, we included only rural areas from Southern Transylvania that are less exploited, thus excluding all known or developed rural areas.

This study aims to: a)identify a decision-making model algorithm applied at local level to support rural development through cultural heritage capitalization (less known on the international market of cultural consumption or for newly discovered cultural heritage assets), based on the improved AHP method; b) to identify the most valuable alternative from a set of strategic alternatives for rural development identified at the regional level. Using a collaborative-approach, our study evaluates the potential of rural development starting from a set of criteria, considered to be important by local stakeholders. Several late researches identified the multiple potential of the decision- making process’s method in different industries [23, 24] or decision levels [25]. By involving local stakeholders, the AHP method allows a partnership approach and based on the results obtained, can enforce innovative business as part of the local strategic development agenda for rural areas.

The present work’s contribution is also theoretically–the development of the AHP model, applicable in the area of heritage capitalization, can contribute to an appropriate decision-making algorithm development. To substantiate our theoretical approach, we conducted a pilot study for less known rural areas located in Sothern Transylvania, based on cultural potential and characterized by a combination of built heritage and natural assets with a rich local intangible heritage (life habits, craft traditions, construction methods that capitalize on local material and allow the promotion of ecological constructions).

Based on the extension of the decision tree, this study also provides a developed version of the AHP method by including particular aspects of local interest and/or relevance. The developed version allows for broader investigation of the defining characteristics that can lead to the development of a trademark associated with the ongoing demand model for cultural heritage capitalization in the context of integrated innovative tourism products in Southern Transylvania. The added value of promoting local authenticity and developing branding on the visibility, attractiveness and economic efficiency of tourism services associated with cultural consumption was investigated using the experts’ opinion on the developed AHP model.

Furthermore, we intended to examine whether the existence of notorious exploitation models of the built heritage (or already recognized as trademarks such as Bran and Peles Castles, Viscri, or the UNESCO enlisted Transylvanian churches) in the vicinity increase the potential for rural tourism and economic and social development of the areas included in this study.

## 2. Literature review

The 19^th^ century contributed to the reintroduction of the rural tourism concept, using the long-distance railways network as a link between the main cities of the continents. Following the Second World War, the concept of rural tourism has expanded, throughout the world [26]. Development through the diversification of economic activities in the area can redefine the social progress and cultural identity models by introducing new concepts and roles that contribute to high quality life in communities [27]. Tourist activities in rural areas are associated with rapid local economic development [28–33].

Several dimensions of culture are associated with tourism, each of which contributes to the economic development of the community [34]. Since the early 1970s, UNESCO has developed and promoted international frameworks for the protection and conservation of cultural heritage, both tangible (buildings, pieces of art, etc.) and intangible (traditional dances and songs, customs and traditions, landscapes natural and cultural), also known as traditional cultural expression [35]. Cultural heritage can contribute to economic development of a region through tourism and cultural economy [36]. TCEs are well debated in the literature around the world. Moreover, the link between them and intellectual property is the focus of most previous research. A recent study critically examines the interaction between TCEs cohesion and European Union trademark law [37]. Multiple debates are held about TCEs and intellectual property rights, especially on trademark matters [38–42]. Preserving intangible heritage or traditional cultural expressions (such as life habits, craft traditions, traditional dance and singing, etc.) as part of cultural heritage is the local community’s social responsibility. It is also a way to enhance rural communities’ development as demonstrated by several good practices. Finally, brands support competitiveness and increase attractivity for consumption. The relationship between local brand and tourism was presented as: local food and tourism by [43, 44], rural cultural heritage by [45–47], food tourism by [48–50], culinary tourism by [51–53], wine tourism by [54–56], agro-tourism by [57–62]. However, the specificity of rural communities requires an entrepreneurial approach adapted to the community’s potential and the opportunities offered by the area [63].

Transylvania has a rich cultural heritage, which contributes nationally to the development of tourism [64, 65]. Probably the most famous historical monument of Transylvania is Bran Castle, which has become an internationally recognized trademark very often visited by tourists (national, but especially international) and which has significantly contributed to the development of the local economy [66]. Visiting the Bran Castles and the Peles Castle (built at the end of the 19^th^ century during the reign of Carol I of Romania, Prince of Hohenzollern-Sigmaringen, belonging to the Royal Family of Romania), was included in almost all tour packages in Romania [67]. Although Southern Transylvania has only partially exploited its cultural heritage, it presents successful innovative entrepreneurship models in rural areas, by incorporating community-based tourism in their economic activities [68]. One of the best-known examples of such models is Viscri, a small village which attracts over 30,000 tourists every year [69]. It is known for the contribution that Prince Charles of Wales had brought to local development through the acquisition of a Saxon household in the village, the restoration and promotion of local cultural heritage, thus attracting in the area many national and international tourists.

Over the years, researchers have concentrated their work on theories that prioritize the conservation, restoration, and rehabilitation of cultural heritage sites by using different methods of multi-criteria decisional analysis [70–74]. Moreover, in the last years, specialists in studies on determining the rural potential development based on cultural heritage, or for defining the investments choice that lead to the development of the local community [75], or the preservation of cultural heritage and identity [76], increasingly used Multi Criteria Decision Making methods. The trademark retrieval systems were created by [77] using AHP methods that classify the information systems’ organizational memory. In the evaluation of the entrepreneurship projects, the AHP method was applied to determine the factors that should be used in the technological projects’ evaluation by [78]. Castaldi in [79] and Flikkema in [80] identified several opportunities for new metrics in terms of trademark value development.

## 3. Data

Data field collection was carried out in 2019, during which working visits were made within the multidisciplinary PORT Cultural project - http://portcultural.usv.ro –, in order to define additional instruments for supporting strategic planning in rural areas with heritage trademark potential.

The theoretical model of decision analysis development has been tested in rural areas located in Southern Transylvania. In the first stage of our investigation, we identified more than 100 potential heritage-built assets that can be capitalized and for which trademarks can be developed hence analyzing, from a monographic perspective, the potential of the associated areas—the rural localities/villages. The selection also considered the (co)existence of intangible heritage elements, that could power the cultural and tourist consumption attractiveness of a wider variety of consumers / tourists, without depriving the local authenticity. Demographic, economic and social factors were selected for monograph development. The 100 built heritage identified in rural areas were documented using photographs and were located in space, and the owners were interviewed, after which they completed a questionnaire consisting of several sections (for example: the construction of the house, but also the social status and economic development of the owners). The local heritage components that define each area were selected, while considering: the European history intrinsic value, the state of cultural heritage conservation assets, specific cultural diversity, local traditions and crafts, natural landscape and other intangible heritage elements.

In the second stage of the field research, in order to evaluate the potential of exploiting existing heritage through tourism, the following aspects were considered: access to road, rail and sanitary infrastructure, number of tourists in the area, possible available accommodation facilities, recognized tourist attractions, the specific cultural activities of the area, etc. Based on the international visibility of the new patrimony objectives and the development/completion of the cultural and thematic routes, we also analyzed the existence in area or in the near vicinity of the recognized heritage brands or trademarks (i.e. UNESCO enlisted, like Sighisoara citadel or Alba Carolina Fortress).

As the research focused on the heritage assets in rural areas, the natural potential was identified hence its status has been evaluated—the level of human intervention/alteration of the habitats, the air quality reported in the area, the presence or proximity with natural parks/reserves and the existence of natural elements of uniqueness (i.e. the steppe peony, the Carpathian bear, etc.). In this study we excluded the rural areas already presenting innovative entrepreneurship models and those developed from a tourist, economic and social perspective (i.e. Viscri village, the Saxon listed villages). However, these represented reference points and models of good practices in the subsequent research in defining the model/profile of entrepreneurial development and designing the integrated local strategy.

Data on the number of tourists and the accommodation capacity of the analyzed areas were selected from the official statistical database (NIS on-line database). The annual reports of the National Environmental Guard provided data on air quality. The specific cultural activities and hospitality infrastructure (i.e. restaurants, accommodation facilities type and details) were extracted using TripAdvisor™ and Booking™ sites. Specific data were also obtained with the support of the local public authorities.

Additional criteria for the inclusion/exclusion of the researched areas namely the level of entrepreneurial development in rural areas (the presence of local stakeholders or those with businesses on complementary services in the area services), the level of accessibility and the existence of other recognized forms of tourism or rural tourism development potential (i.e. tourism for health, mountain circuits or traditional sports competitions, artistic creation camps, etc.) were considered. Using the AHP method, many researchers applied similar criteria on cultural heritage/ cultural tourism assessment [81–84].

Following the collection of these data, we established several areas of interest for our multi-criteria decision-making analysis, both geographically and socio-economically similar. Rural areas with similar characteristics and with close geographical proximity have been grouped into clusters. These clusters form the 16 alternatives included in our research and are briefly described in Table 1 and S1 Appendix (attached as supplemental material [85]). We also used the aforementioned quantitative collected data to assign the evaluation criteria for the AHP method. Supplemental materials attached to this work provide information on the method application and the alternatives and criteria identified throughout data collection process.

**Table 1:** A description of the alternatives selected for multi-criteria analysis.

Alternatives	Rural areas specific elements (clusters`main characteristics)
**Alternative**_**1**_	Mountain area, rich in the national interest’s complex natural landscapes. Nature reserves listed in Natura 2000; protected areas, unique in Europe. The basic activities consist mainly in natural tourist routes. Diversified built heritage (traditional houses, churches, etc.)
**Alternative**_**2**_	Mountain area, rich in natural and cultural landscapes. Geographic features are similar to those in alternative 1. The area is famous due to “Awake” electronic music festival but also for built cultural heritage (masons, churches, castles, etc.) and for cultural landscape.
**Alternative**_**3**_	Hill area, famous for Steppe Peony Natural Reservation, unique in Europe by being located at the highest altitude. Multiple tourist activities (fishing, horse riding, cycling, hiking trails). Diversified built heritage.
**Alternative**_**4**_	Area incorporating an airport, known for built cultural heritage (particularly references), having historical importance of national interest.
**Alternative**_**5**_	Mountain area, rich in natural landscapes, limestone formations, forming an area of extensive and spectacular gorges. Nature protected areas listed in Natura 2000, unique in Europe. It is in the vicinity of the Székely village Rimetea, which was awarded for conservation of the built heritage, being subsequently enlisted in the UNESCO World Heritage.
**Alternative**_**6**_	Mountain area with a rich historical past. Trademark is given by the viniculture and wine cellars recognized. Tourist attractions: tourist routes to various limestone formations (the longest, deepen cave in Romania, keys, gorges), the historical monuments; leisure activities (cycling, rafting, paragliding, etc.)
**Alternative**_**7**_	Historic area, rich in multicultural landscape. Villages which combine architectural elements from Romanian and Saxon culture. Historical monuments of national interest; natural and geological reserves; saltwater springs. Sights: intangible heritage, tangible heritage and Dacian baths, Via Transilvanica cultural route, etc.
**Alternative**_**8**_	Historical area, inhabited by Romanians, Saxons and Hungarians. Numerous historical monuments; tourist attractions: countryside Bazna resort, castles, wine cellars (Cetatea de Baltă, Jidvei), fortified churches, local restaurants etc. Tourism based on experience, tourist live unique emotional experiences, retracing the life of the countryside.
**Alternative**_**9**_	Mountain area mostly inhabited by Saxons. Sights: historical monuments, traditional cuisine, festivals, cultural events, travel, music festivals, ski resorts, etc.
**Alternative**_**10**_	Area situated in the vicinity of the spectacular Transfăgărășan road, which offers its guests a variety of services and tourism facilities (travel, hiking, skiing, historical monuments, festivals, climate, resort castles, etc.). It incorporates the geographical center of Romania.
**Alternative**_**11**_	Relatively hard accessible area, located in a spectacular natural landscape, near Bran and Peleș Castles. Rural and natural tourism very developed; numerous recreational activities. Numerous historical and cultural monuments (fortresses, fortified churches, monasteries, Dacian, roman castrum, archaeological site, etc.). Annually, this area houses a rock music festival.
**Alternative**_**12**_	Area with numerous historic monuments; rural complex "wooden houses"; nature reserves, mineral water springs. Numerous Hungarians and Székely cultural activities.
**Alternative**_**13**_	Mountain area with numerous unique or rare landforms in Romania. Tangible and intangible cultural heritage highly diversified. Developed tourism on several branches (based on natural experiments, seasonal, etc.).
**Alternative**_**14**_	Culturally diversified area, easily accessible, nationally recognized for its innovative business models, its associated travel forms and various possibilities of leisure activities under tourist attractions (facilities with salt water, rafting, e-bike travel-service, salt mine, nature reserves, museums, pottery workshops, adventure park, equestrian, ski resort, fishing, etc.). Area with high level of accessibility in terms of infrastructure. Rural areas (the Székely and Saxon) which develop niche tourism, such as tourism based on experience.
**Alternative**_**15**_	Mountain area with numerous natural reserves; diversified cultural heritage; multiple possibilities of hiking trails and recreational activities.
**Alternative**_**16**_	Area of national interest, both in terms of geographical and historical facts. Numerous tourist resorts with recreational, tourist or cultural activities. Traditional festivals, mineral water springs, spectacular limestone formations, historical monuments, etc.

(source: authors own research)

**Fig 1:**
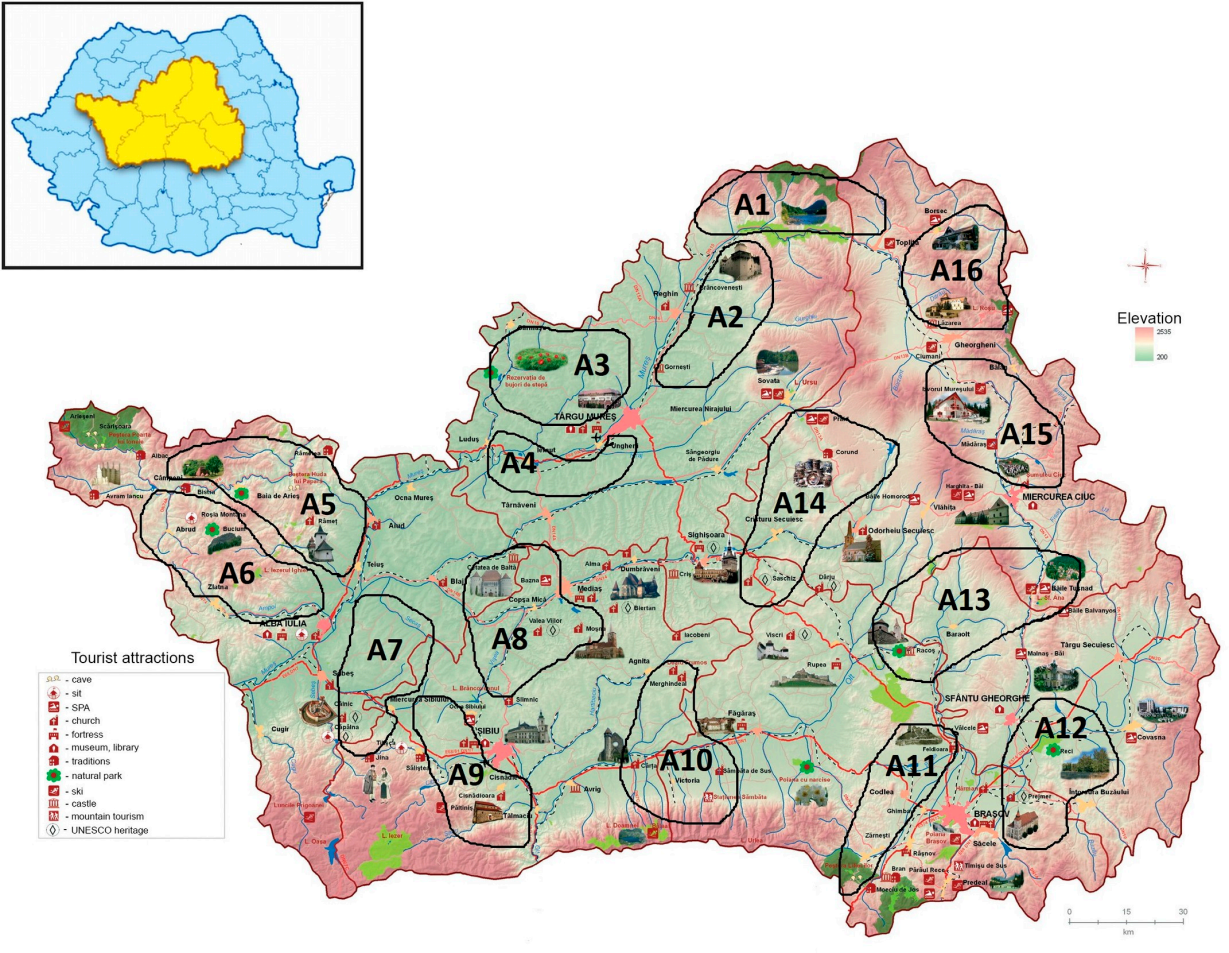
Geographic localization of selected alternatives. Source: Authors’ projection using the maps reprinted from ADR under a CC BY license, with permission from ADR Centru, original copyright 2016.

All the 16 selected alternatives contain rural areas that are characterized by a complex and multicultural landscape that incorporates cultural heritage elements (tangible and intangible) with an increased potential in trademark creation. The alternatives were chosen by the experts from the project research team, based on the analysis of the data described in Table 1 and S1 Appendix, as shown in Fig 1.

In our approach to identify and prioritize the alternatives for strategic rural development through innovative heritage entrepreneurship, several criteria were chosen with the help of the data collection process. During the working meetings and with specialists, we conducted several brainstorming sessions, for the selection of the main criteria needed to apply the multi-criteria decision analysis, based on the developed AHP method. These main criteria refer to the area’s **recognized brands**, the level of **accessibilit**y, the classification of the areas in the **natural landscape**, the existence of **cultural heritage** as well as the **facilities** offered by the selected alternatives. The criteria’s description can be identified in S2 Appendix, and [85].

## 4. Research methodology

AHP has multiple applications in optimal allocation of resources. It has been shown particularly useful in urban or rural valorization process and development process, as a tool of mediation among multiple and sometimes divergent interests and it is able to create a shared platform among decision makers and stakeholders directly affected by the final decision. AHP deals both with the problems of efficiency and equity of choices. In this sense, AHP is a flexible instrument to compare values and qualitative aspects of decisions considering all various aspects of a problem. It integrates knowledge of different fields of expertise, considering the complexity of the context and the consequences of every alternative option.

Using the AHP multi-criteria decision-making method, we identified the most suitable area for rural development through innovative entrepreneurship by highlighting the cultural heritage in the rural area, with potential for a trademark design. In summary, the research was structured in the following steps:

***Step 1*:** Defining the main purpose (main goal) of the research and selecting the outcomes.
***Step 2*:** Conducting field study visits and developing monographs for each of the initial selected areas, organizing qualitative research based on focus groups and in-depth interviews, brainstorming sessions with the project research team and literature review in the field. Based on the results obtained in these activities, the evaluation criteria and their importance were established from trademark creation and strategic rural development perspectives.
***Step 3*:** For accurate research results, we collaborated with experts in the reconditioning cultural heritage, the landscape and natural sciences, the rural entrepreneurial development, the behavioral theory of the consumer and tourism economics. The experts were selected based on the discussions within the focus groups, their independence and activeness in the business environment. The selected experts independently analyzed and evaluated the specific established criterion having the freedom to assign and assess sub-criteria where appropriate, based on professional experience and reasoning.

Expert 1 –selected for the evaluation of *Trademark* criterion–is the Brand manager in a national based company, specialized in branding strategy or brand story design, identifying the main target group of the brand and the company's core values, building the brand positioning and communication strategy for the market. Using professional skills and rationing, expert 1 established 5 sub-criteria for the Trademark criterion and weighted an importance for each sub-criterion. For example, expert 1 considers that the trademark for local tourism development is perceived differently depending on the profile of visitors, the presence and promotion in the online environment of recognized tourist objectives, the authenticity of experiences given by visiting trademarked places. Therefore, these are some of the sub-criteria assigned by the expert to the trademark criterion.

Expert 2 –selected for the *Accessibility* criterion analysis–is an independent expert, self-employed specialist in the economic impact and counterfactual analysis, spatial development, rural entrepreneurship and sustainable growth of the small areas. Expert 2 considers that an objective can become of tourist interest if it is located in an area with high accessibility, for example close to main roads, railways or airport. However, from the professional experience, the expert considers that, on the contrary, a certain type of tourists visits areas that are not easily accessible, out of the desire to disconnect from work and their normal life. Therefore, two sub-criteria were considered relevant by the expert, namely: easy access and remote. These two sub-criteria for the Accessibility criterion and weighted an importance to each one.

Expert 3 –chosen for the evaluation of the *Landscape* criterion–is an architect specialized in landscape, urbanism and spatial planning, sustainable development, urban and rural revitalization, mobility, capitalization of natural and built heritage and energy efficiency of new and old buildings, being a member of a specialized NGO in Built Environment, active in Southern Transylvania, with multiple projects to enhance the natural environment and its integration in cultural and leisure routes. In the context of the potential for tourism development, given the potential profile of tourists, expert 3 considers that a rural area can be developed for tourism if the area is recognized for good air quality, natural beauty (eg nature reserves) or other attractions. travel. Therefore, expert 3 established 3 sub-criteria for the Landscape criterion and weighted an importance to each one.

Expert 4 –selected for the *Cultural Heritage* criterion–is an economic expert in cultural heritage capitalization. They were a national expert for the Ministry of Culture from Romania and the National project manager for INE, in THE JOP BLACK SEA CBC ALECTOR Collaborative PROJECT Multilevel networks of Actors, to advance Quality Standards for Heritage Tourism at Cross Border Level. Expert 4 was also the National Project Manager for Launching local level entrepreneurship heritage: strategies and tools to safeguard the United forces, like cultural values, mobilize, and deliver the experience (SAGITTARIUS). SEE schedule, with Certified EQF 06, expert in heritage interpretation. Expert 4 did not consider it necessary to assign sub-criteria for the Cultural Heritage criterion, considering however, that this main criterion must be highly important for the analysis performed using the multi criteria analysis method.

Expert 5 –selected for the *Facilities* criterion–is a specialist in tourism economics and innovative hubs development Agency and is the promoting international tourism marketing manager for integrated hospitality, specialized in the incoming activities for the tourist’s International member EVANEOS. The expert considers that already existing accommodation facilities, restaurants and tourist activities in one area can contribute to rural development. Thus, expert 5 assigned 3 sub-criteria for the Facilities criterion and weighted an importance to each one.

***Step 4*:** Application of the AHP method according to the methodology developed by Saaty [86], and the best alternative determination for rural strategic development through innovative entrepreneurship in the areas identified. In addition to Saaty’s model, we developed the decision tree with supplementary (sub) criteria, while refining the research’s outcomes.

The criteria selection method, which was based on both quantitative and qualitative approaches, started from statistical evidence and evaluating identified heritage potential in each area, as described in Section 2.

Simkova [87] describes the procedures used for analyzing the rural tourism development’s potential, in order to assess the most suitable area for investments, while considering demographic factors, social and economic aspects, plans for local communities, the area’s potential analysis (natural, cultural, social and economic potential) and its current status analysis (expressed by the level of attractiveness, accessibility to funding sources, the pollution level, etc.). Similar to these remarks, we identified several rural areas with high potential for tourism development, based on innovative entrepreneurship models in the context of climate and sustainable development for everybody [88] and Agenda 2030 [89] from United Nations.

After the development of the monographs in the research area, we identified and selected the localities/villages with the highest potential for rural heritage trademark creation and capitalization. In addition to the components mentioned by Simkova in [87], we selected the accessibility level as the evaluation criteria (also studied by [90]), i.e. the existence of additional facilities—accommodation, gastronomic tradition, tourist routes, specific cultural events or other tourist activities (also studied by [91, 92]).

Three qualitative methods were used to identify the analysis criteria and to substantiate their choice i.e. a qualitative survey based on defined questionnaire applied to the owners of the 100 built heritage assets identified in the area and eight focus groups with local stakeholders, including public authorities (as shown in Table 2). Subsequently, based on the results obtained through focus-groups, 20 in-depth interviews were conducted for relevant local entrepreneurs in cultural heritage capitalization.

**Table 2:** Stakeholders’ participants at focus groups.

	Self-employed and family associations	SMEs’	NGO	Public authorities
**Focus groups**	10	18	12	8
**In-depth interviews with local active entrepreneurs**	2	10	8	3

(Source: PORT Cultural project database)

The focus groups meetings and in-depth interviews revealed issues related to the need for investment in tourism development in rural areas. The local entrepreneurs present at the focus groups emphasized the importance of "smart" investments. Thus, they mentioned certain critical aspects that would motivate them to invest in a certain location to create a tourism business. Most said that the nearby existence of cultural heritage or natural tourist attractions recognized at least nationally is essential for the investment decision. For example, one of the participants stated:

*"The development of Viscri is also felt in the immediate vicinity and the increasing inflow of tourists in that village impacts the tourism and economic development in the surrounding areas*.*" (D.S.–local entrepreneur and business owner)*

Existing facilities in the area were discussed to be important for their investment decisions. In addition to the attractiveness of the area, most said that infrastructure is also important, both road, air or rail, and health. Others believed that there is a need for a quiet, authentic area with little impact on the environment, which has not undergone significant changes due to technological developments to develop rural cultural tourism. All participants were of the opinion that cultural tourism is frequented by a certain category of people, being considered a form of niche tourism. Participants also stated that they are willing to invest in rural cultural tourism by creating innovative and sustainable business models. One of the participants stated:

*"If I knew exactly where I should place my tourism business*, *I would implement a locally innovative idea to create a successful sustainable business model that would bring fame to the area and stimulate the local economy*.*" (B.M.–Cultural Heritage Capitalization NGO leader)*

Based on the preliminary results of the on-site meetings with local entrepreneurs, we built an AHP model using the selected criteria: trademark potential, accessibility, landscape, existent cultural heritage capitalization potential and facilities. Each criterion was assigned to a collaborating expert for further analysis. The experts created sub-criteria where they considered and established the importance (according to Saaty scale [86]) of each sub-criterion by comparison, building comparison matrices.

**Fig 2:**
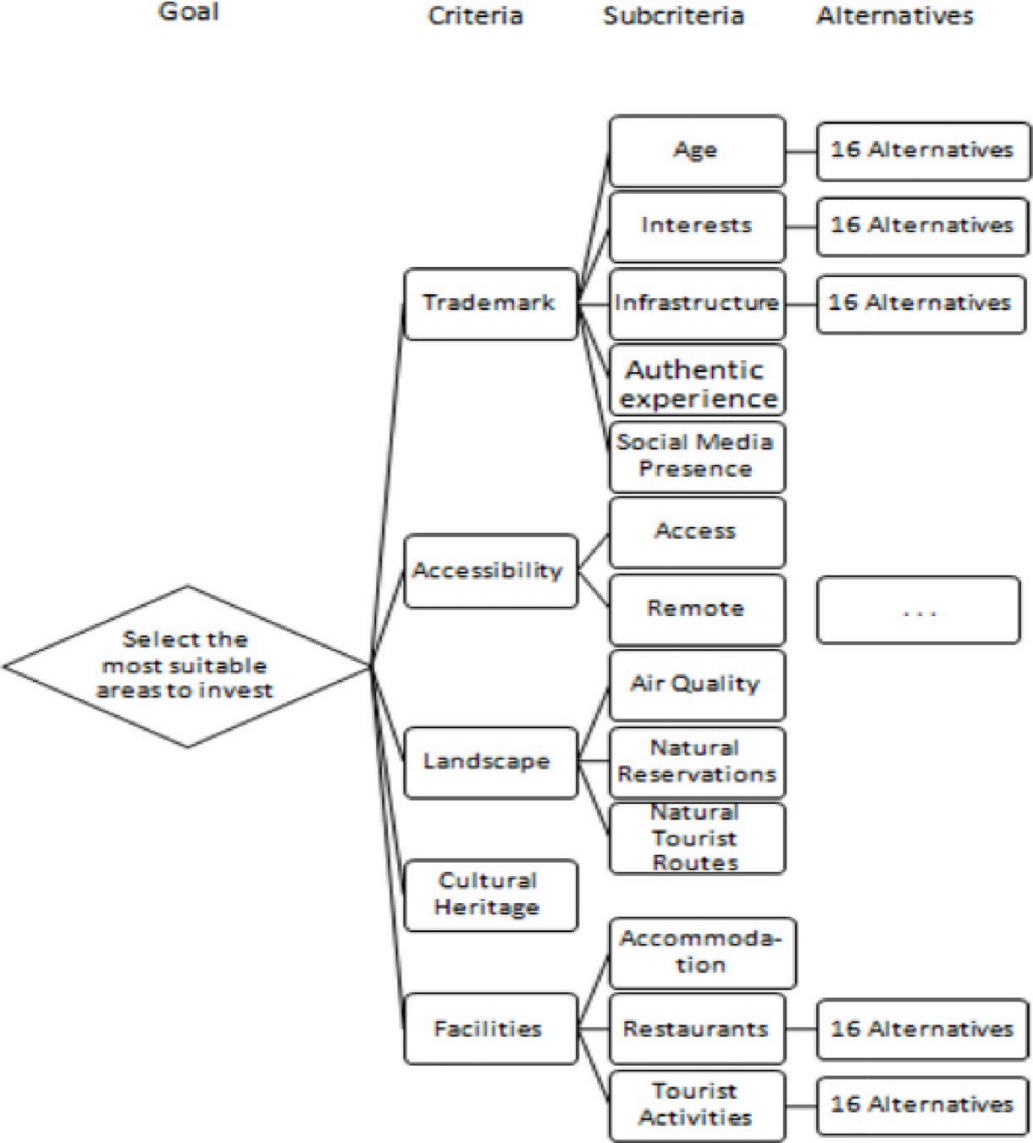
A three-level criteria tree with five criteria each with sub-criteria developed to select the most suitable investments’ area in South Transylvania based on cultural heritage and trademark potential. (Source: Authors’ own projection).

The pairwise comparison method was first introduced into research by Fechner [93]. Thurstone in 1927 developed the initial form of the AHP method [94], in order to describe a law of comparative judgment. The AHP approach, developed by Saaty [95–97] represents a Decision Support System (DSS) based on Multi-Criteria Decision-Making (MCDM) methods for analyzing complex decisions. Our decisional tree was structured as follows: *goal*, *criteria*, *sub-criteria*, and *alternatives selection*. We developed the AHP model version, with three levels as shown in Fig 2.

Methodologically, the AHP approach’s goal is the general objective that drives the DSS problem, whereas *alternatives* are the different options being weighted in the decision. *Criteria* and *sub-criteria* are the factors used to evaluate the alternatives regarding the main goal (using the Saaty scale as described in Table 3).

**Table 3:** Table of relative scores.

Value of *x*_*ij*_	Interpretation
**1**	*i* and *j* are equally important
**3**	*i* is slightly more important than *j*
**5**	*i* is more important than *j*
**7**	*i* is strongly more important than *j*
**9**	*i* is absolutely more important than *j*
**2,4,6,8**	intermediate values between two judgments

(source: Saaty [86])

AHP model was built using pairwise comparison matrices, which can generally be presented as a *x*_*ij*_ matrix, in the form of:

$$X={\left(x\right)}_{ij}=\left[\begin{array}{cccc} x_{11} & x_{12} & \cdots & x_{1n}\\ x_{21} & x_{22} & \cdots & x_{2n}\\ \vdots & \vdots & \ddots & \vdots \\ x_{n1} & x_{n2} & \cdots & x_{nn} \end{array}\right]=\left[\begin{array}{cccc} \frac{w_{1}}{w_{1}} & \frac{w_{1}}{w_{2}} & \cdots & \frac{w_{1}}{w_{n}}\\ \frac{w_{2}}{w_{1}} & \frac{w_{2}}{w_{2}} & \cdots & \frac{w_{2}}{w_{n}}\\ \vdots & \vdots & \ddots & \vdots \\ \frac{w_{n}}{w_{1}} & \frac{w_{n}}{w_{2}} & \cdots & \frac{w_{n}}{w_{n}} \end{array}\right]=\left[\begin{array}{cccc} 1 & \frac{w_{1}}{w_{2}} & \cdots & \frac{w_{1}}{w_{n}}\\ \frac{w_{2}}{w_{1}} & 1 & \cdots & \frac{w_{2}}{w_{n}}\\ \vdots & \vdots & \ddots & \vdots \\ \frac{w_{n}}{w_{1}} & \frac{w_{n}}{w_{2}} & \cdots & 1 \end{array}\right]$$ (1),

with $x_{ii}=1,x_{ij}=\frac{w_{i}}{w_{j}}=\frac{1}{x_{ji}},$ *x*_*ij*_ used values from Saaty scale (see Table 3), where *x*_*ij*_−is the relative importance of criterion *i* compared with criterion *j*, respectively, *x*_*ji*_ − represents the relative importance of criterion *j* compared with criterion *i* and

$$\sum_{i=1}^{n}w_{i}=1$$ (1.1)

The matrices of comparisons are used to compare criteria, sub-criteria and alternatives.

A matrix *X* is ideally consistent if the relationship between all the elements is similar. But due to the limitation of human-being to assign ideal quantification in case of complex decision-making problems (a large number of alternatives, for example), testing the consistency of comparison matrices becomes extremely important for establishing viable final results. However, Saaty has introduced indicators for calculating the consistency of a matrix, called consistency index (CI) and consistency ratio (CR), which allow the consistency of the matrices developed in the decision-making process to be checked, given the human errors that may occur. He considered the CR level at a maximum of 0.10 to be acceptable, which means that the comparison matrix must be at least 90% consistent. In order to determine CR, first we had to determine CI (using the largest eigenvalue as Saaty defined in the methodology) and Random Index (*RI*). We used the values of *RI* for our analysis as they were calculated by [98].

If the pairwise comparison matrices were consistent (*CR*<0.10), we normalized the values in order to homogenize the data obtained through initial evaluation. The general normalized matrix $\bar{X}$ is computed as:

$$\bar{X}={\left(\bar{x}\right)}_{ij}=\left[\begin{array}{cccc} \frac{x_{11}}{\sum_{i=1}^{n}x_{i1}} & \frac{x_{12}}{\sum_{i=1}^{n}x_{i2}} & \cdots & \frac{x_{1n}}{\sum_{i=1}^{n}x_{in}}\\ \frac{x_{21}}{\sum_{i=1}^{n}x_{i1}} & \frac{x_{22}}{\sum_{i=1}^{n}x_{i2}} & \cdots & \frac{x_{2n}}{\sum_{i=1}^{n}x_{in}}\\ \vdots & \vdots & \ddots & \vdots \\ \frac{x_{n1}}{\sum_{i=1}^{n}x_{i1}} & \frac{x_{n2}}{\sum_{i=1}^{n}x_{i2}} & \cdots & \frac{x_{nn}}{\sum_{i=1}^{n}x_{in}} \end{array}\right]$$ (2)

Using the arithmetic mean of each line of the normalized matrix, we obtained the scores of each criterion, sub-criterion and alternative. In the end, we built the decision matrix, in which we passed the scores obtained through normalization. Then we multiplied the scores obtained by each sub-criterion (or criterion) with those obtained by each alternative. The final results (overall priorities) are represented by the sum of the scores obtained by multiplication of each line (represented by each alternative). For an easier interpretation of the results, we ordered the alternatives depending on the score obtained. Thus, the alternative with the highest score obtained ranks 1^st^ in the ranking, while the alternative with the lowest score obtained ranks 16^th^ in the ranking (see S4 Appendix and Table 5).

## 5. Results and discussion

### 5.1 Criteria and sub-criteria priorities

To determine the best alternative for investing in the development of cultural tourism in rural areas, we used five criteria that local stakeholders (present at meetings held in the first stage of research) mentioned as basic for investing in business models in the field of tourism. Therefore, our analysis responds to the needs of entrepreneurs involved in tourism industry of Southern Transylvania rural areas. Criteria and sub-criteria priorities, as well as their rank are found in Table 4. The criteria matrix (see S2–S4 Appendices, attached as supplemental material [85]) was composed by the research team as a result of qualitative research performed through brainstorming sessions during the meetings organized in the project’s framework, on account of the questionnaires application, focus group results and in-depth interviews.

**Table 4:** Criteria and sub-criteria ranking by priorities.

Criteria	Priorities	Criteria Rank	Sub-criteria	CR	Priorities	Sub-criteria Rank
**Trademark**	0,316	1	Age *(SC*_*11*_*)*	0,04	0.021	12
			Interest *(SC*_*12*_*)*		0.104	2
			Infrastructure *(SC*_*13*_*)*		0.045	10
			Authentic experience *(SC*_*14*_*)*		0.060	6
			Presence in social media *(SC*_*15*_*)*		0.085	3
**Accessibility**	0,058	5	Easy access *(SC*_*21*_*)*	0	0.049	9
			Remote *(SC*_*22*_*)*		0.010	13
**Landscape**	0,129	4	Air quality *(SC*_*31*_*)*	0	0.051	7
			Natural reservations *(SC*_*32*_*)*		0.051	7
			Tourist attractions *(SC*_*33*_*)*		0.026	11
**Cultural Heritage**	0,205	3	-	N/A	-	-
**Facilities**	0,292	2	Accommodation *(SC*_*51*_*)*	0	0.073	4
			Restaurants *(SC*_*52*_*)*		0.073	4
			Touristic activities *(SC*_*53*_*)*		0.146	1

(Source: Authors’ own calculation)

The *Trademark* criterion obtained the highest score, with an importance of over 30%. It is followed by the *Facilities* criterion, *Cultural Heritage*, *Landscape*, and *Accessibility*, respectively. The sub-criteria rank was established by comparing each sub-criteria pair established by the independent experts (see Table 4 and S4 Appendix).

Collaborating with independent experts added value to our research, by assigning some sub-criteria identified to be relevant for a more in-depth analysis, leading to the achievement of relevant results, with immediate practical applicability. The experts’ professional judgment considers the touristic activities existing in the area of the 16 alternatives a prime sub-criterion (part of *Facilities* criterion), followed by the two sub-criteria given by the *Trademark* criterion (Interest and Presence in social media). The capacity and type of accommodation, as well as the existence of restaurants in the area are also crucial sub-criteria. The criteria and sub-criteria matrix’s consistency ratio is below the acceptable value of 0.10 (see column 5 of Table 4). Based on these results, we made comparisons for each alternative.

### 5.2 Alternatives priorities

For each criterion or sub-criterion, the alternatives were compared two by two through pairwise comparisons while assessing weights, using the proposed Saaty scale [86] (presented in Table 3, Research Methodology section). Thus, we obtained 14 comparison matrices, with a consistency ratio below the acceptable value, 0.10 (see S4 Appendix). Through normalization, we calculated the arithmetic means on every line of the matrix, which represented the priorities obtained by each alternative. Then, as presented in the Research Methodology Section, we multiplied alternatives’ priorities with criteria/sub-criteria’s priorities. With the arithmetic mean of these calculations we obtained the overall priorities of each alternative. These results are summarized in Table 5.

**Table 5:** AHP results.

Criteria	C_1_ Trademark					C_2_ Accessibility		C_3_ Landscape			C_4_ Cultural Heritage	C_5_ Facilities			Overall Priorities
Criteria priorities	0,316					0,058		0,129			0,205	0,292			
**Sub-criteria**	**SC**_**11**_	**SC**_**12**_	**SC**_**13**_	**SC**_**14**_	**SC**_**15**_	**SC**_**21**_	**SC**_**22**_	**SC**_**31**_	**SC**_**32**_	**SC**_**33**_	**-**	**SC**_**51**_	**SC**_**52**_	**SC**_**53**_	
**Sub-criteria priorities**	0,068	0,331	0,144	0,189	0,269	0,833	0,167	0,400	0,400	0,200	-	0,250	0,250	0,500	
**Global priorities**	0,021	0,104	0,045	0,060	0,085	0,049	0,010	0,052	0,052	0,026	0,205	0,073	0,073	0,146	**1,000**
**A**_**1**_	0,058	0,054	0,113	0,056	0,051	0,113	0,079	0,097	0,118	0,116	0,015	0,053	0,023	0,039	**0,054**
**A**_**2**_	0,022	0,028	0,073	0,021	0,090	0,073	0,069	0,046	0,068	0,067	0,052	0,031	0,042	0,038	**0,049**
**A**_**3**_	0,039	0,050	0,036	0,036	0,040	0,036	0,056	0,030	0,033	0,020	0,027	0,015	0,017	0,034	**0,032**
**A**_**4**_	0,040	0,044	0,166	0,038	0,033	0,166	0,109	0,027	0,018	0,014	0,048	0,021	0,041	0,019	**0,047**
**A**_**5**_	0,067	0,067	0,043	0,063	0,059	0,043	0,040	0,055	0,090	0,065	0,055	0,048	0,055	0,070	**0,060**
**A**_**6**_	0,036	0,037	0,021	0,033	0,030	0,021	0,023	0,033	0,049	0,050	0,066	0,030	0,036	0,037	**0,041**
**A**_**7**_	0,044	0,045	0,030	0,052	0,040	0,030	0,034	0,033	0,026	0,020	0,057	0,026	0,028	0,028	**0,039**
**A**_**8**_	0,087	0,084	0,040	0,081	0,085	0,040	0,045	0,054	0,028	0,021	0,158	0,044	0,058	0,070	**0,082**
**A**_**9**_	0,046	0,047	0,050	0,046	0,046	0,050	0,048	0,046	0,059	0,066	0,038	0,071	0,069	0,064	**0,052**
**A**_**10**_	0,055	0,058	0,105	0,055	0,064	0,105	0,082	0,057	0,095	0,112	0,077	0,113	0,117	0,083	**0,082**
**A**_**11**_	0,088	0,088	0,016	0,104	0,080	0,016	0,183	0,151	0,163	0,136	0,017	0,170	0,155	0,133	**0,094**
**A**_**12**_	0,085	0,080	0,069	0,064	0,072	0,069	0,054	0,066	0,034	0,021	0,021	0,028	0,040	0,028	**0,046**
**A**_**13**_	0,069	0,067	0,059	0,082	0,072	0,059	0,044	0,061	0,041	0,071	0,100	0,064	0,073	0,078	**0,074**
**A**_**14**_	0,110	0,109	0,065	0,126	0,103	0,065	0,041	0,069	0,041	0,066	0,161	0,084	0,082	0,084	**0,101**
**A**_**15**_	0,075	0,070	0,053	0,071	0,063	0,053	0,039	0,087	0,064	0,074	0,028	0,104	0,082	0,098	**0,067**
**A**_**16**_	0,078	0,070	0,062	0,071	0,070	0,062	0,054	0,089	0,074	0,082	0,079	0,100	0,082	0,098	**0,080**

(Source: Authors’ own calculation)

Table 5 shows the alternatives’ local priorities for each criterion and/or sub-criterion. The last column presents the overall priorities obtained by each alternative by comparing them while considering the criteria and sub-criteria. They can be ranked according to the overall priorities obtained, thus determining the most suitable alternative for the strategic development based on cultural heritage tourism.

**Fig 3:**
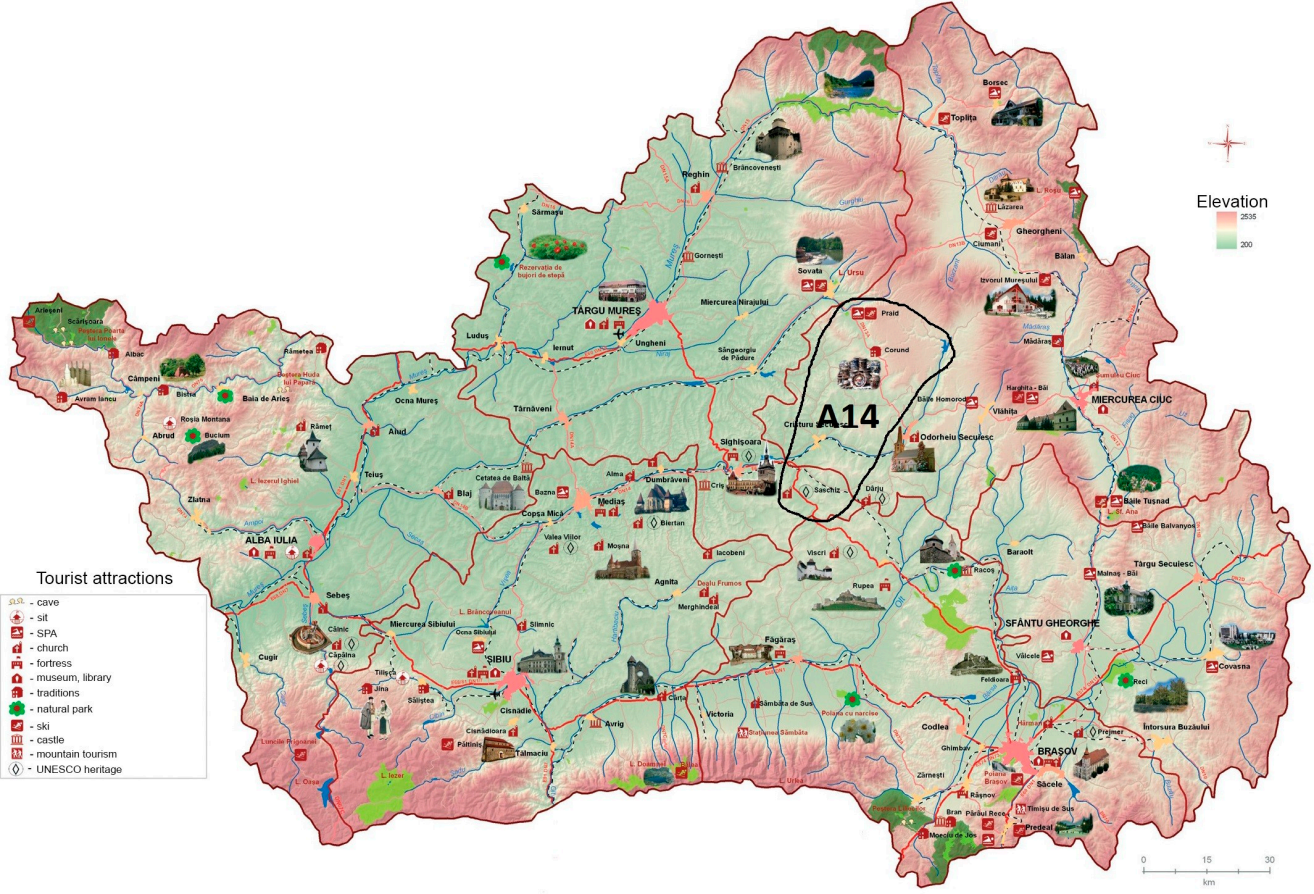
Geographic localization of A_14_. Source: Authors’ projection using the maps reprinted from ADR under a CC BY license, with permission from ADR Centru, original copyright 2016.

**Fig 4:**
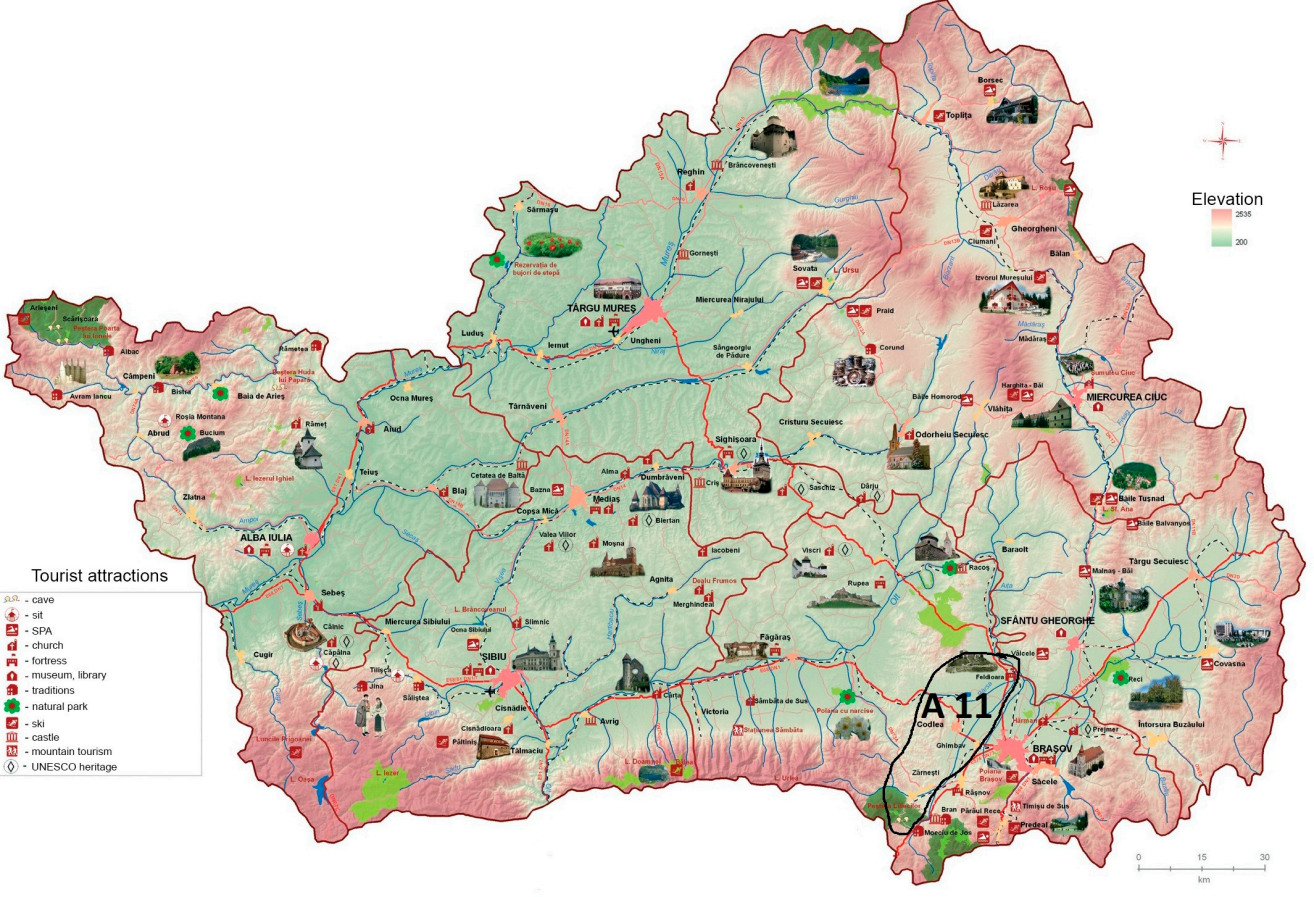
Geographic localization of A_11_. Source: Authors’ projection using the maps reprinted from ADR under a CC BY license, with permission from ADR Centru, original copyright 2016.

We observed that the overall *A*_14_ and *A*_11_ priorities obtained are very close. This is an unexpected result considering their different profiles (see Table 2 in section frame Data and S1 Appendix). However, these alternatives have a common point of reference, as both are located near internationally famous heritage tourist attractions (i.e. Bran and Peles Castle, Viscri, etc). Thus, *A*_14_ (see Fig 3) is located near the Viscri area, a medieval Saxon village enlisted as a UNESCO site with innovative rural and cultural tourism business models. A_11_ on the other hand, (see Fig 4) is located in Rucar-Bran corridor’s vicinity, being famous for Bran Castle, as illustrated in Bram Stoker's novel "Dracula" and for Peles Castle owned by the Royal Family of Romania–King Ferdinand I of Hohenzollern and Queen Marie of Edinburgh. These results show the importance of the trademark elements’ existence in the area or its vicinity for the potential of entrepreneurial development in rural areas. As the results obtained in the first phase of the research show, investments in the heritage tourism development should occur in the vicinity of already recognized tourist locations.

**Fig 5:**
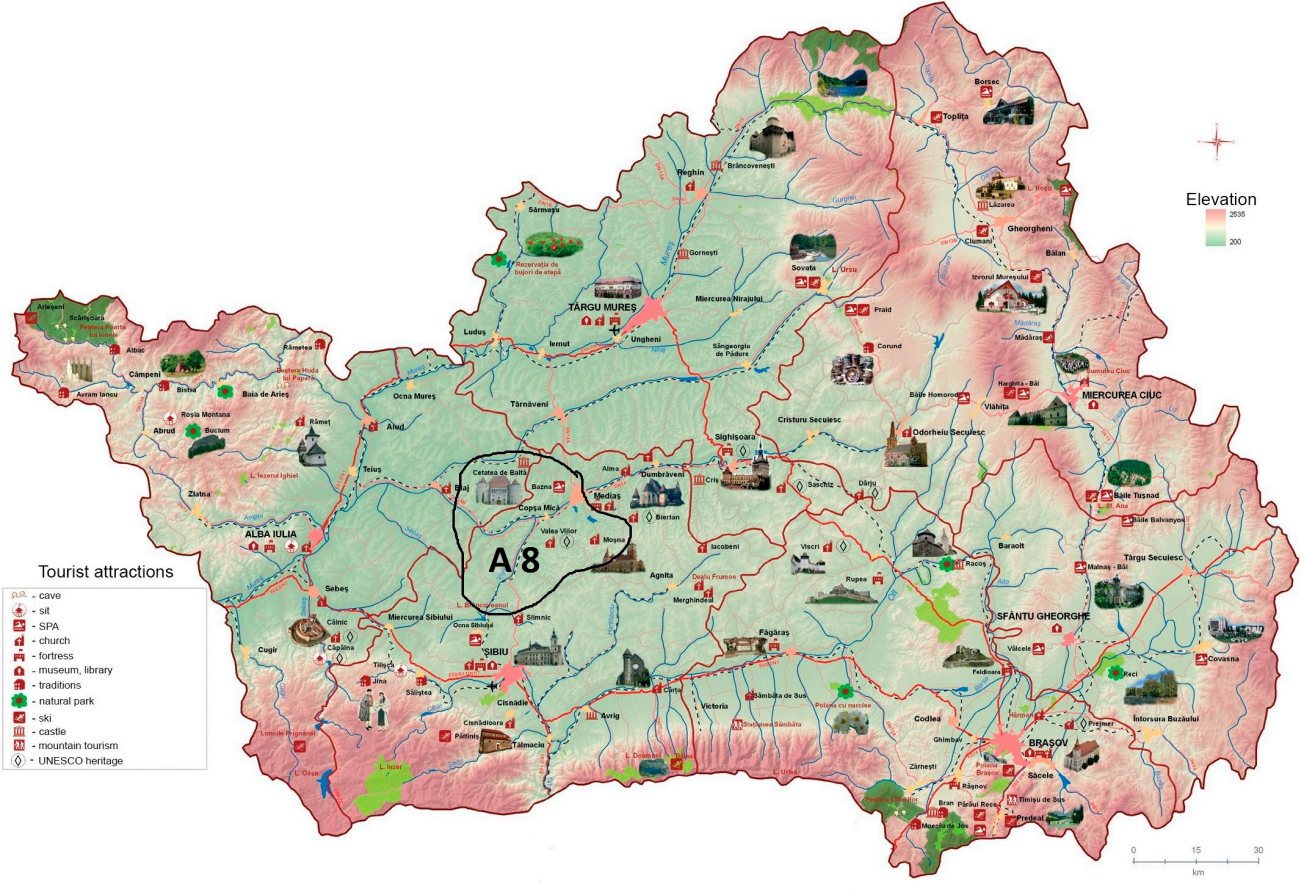
Geographic localization of A_8_. Source: Authors’ projection using the maps reprinted from ADR under a CC BY license, with permission from ADR Centru, original copyright 2016.

**Fig 6:**
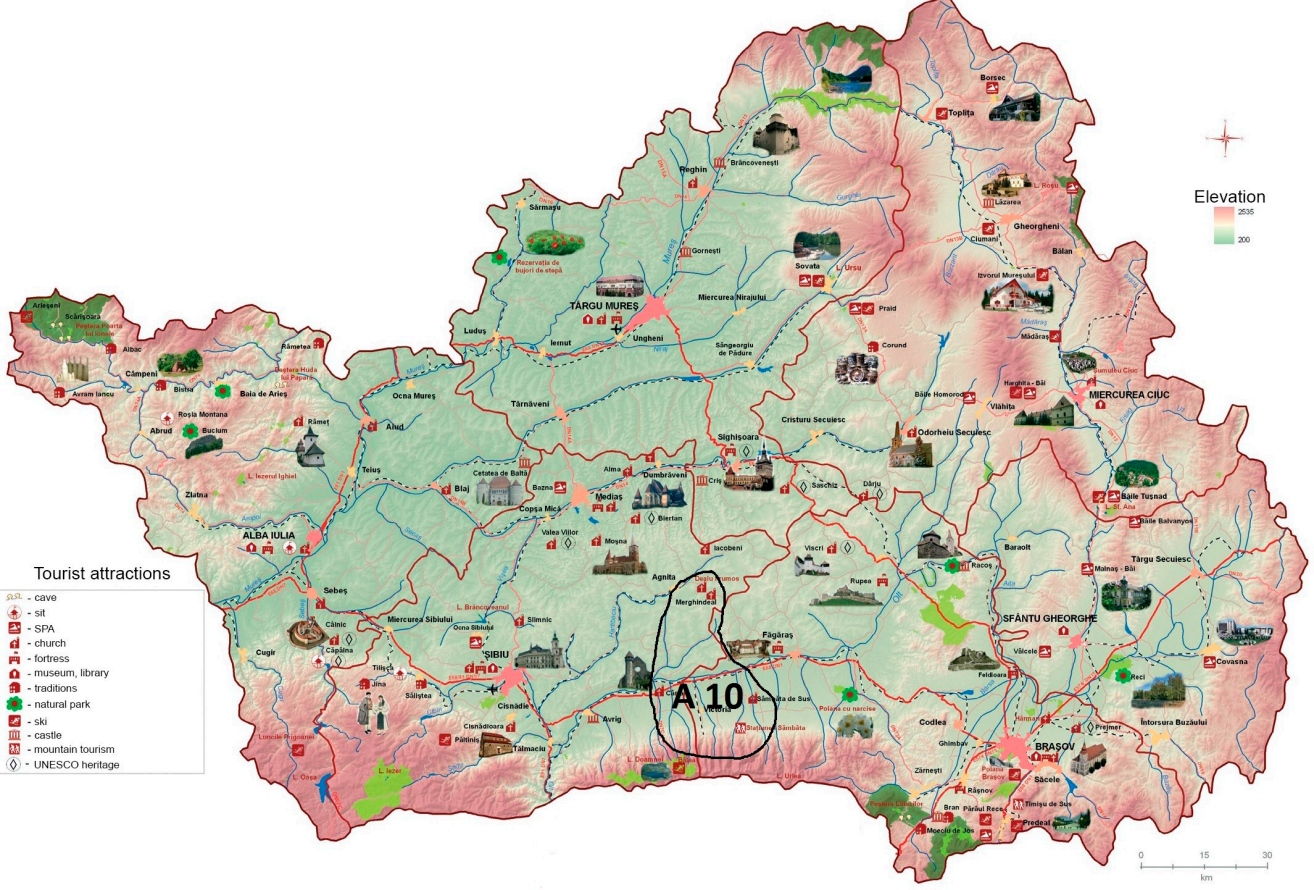
Geographic localization of A_10_. Source: Authors’ projection using the maps reprinted from ADR under a CC BY license, with permission from ADR Centru, original copyright 2016.

Position 3 in the hierarchy of the alternatives is occupied by *A*_8_ (see Fig 5) and *A*_10_ (see Fig 6), whose common point is represented by the trademark elements that can be found in the area or its proximity (Jidvei Castle and winery and the Transfagarasan road at an altitude of 2,042 meters).

Based on the calculations and considering the criteria’s and sub-criteria’s determined values for evaluating the alternatives, our proposed order for the implementation of future strategic development projects is presented in Table 6.

**Table 6:** Alternatives ranking.

Alternatives	A_1_	A_2_	A_3_	A_4_	A_5_	A_6_	A_7_	A_8_	A_9_	A_10_	A_11_	A_12_	A_13_	A_14_	A_15_	A_16_
Rank	9	11	16	12	8	14	15	4	10	3	2	13	6	1	7	5

(Source: Authors’ own calculation).

In another form, the hierarchy of decision alternatives can be presented as follows: *A*_14_≻*A*_11_≻*A*_10_≻*A*_8_≻*A*_16_≻*A*_13_≻*A*_15_≻*A*_5_≻*A*_1_≻*A*_9_≻*A*_2_≻*A*_4_≻*A*_12_≻*A*_6_≻*A*_7_≻*A*_3_. Therefore, the location identified as the most suitable for the strategic development projects in order to increase the trademark potential through heritage-based rural entrepreneurship is represented by *A*_14_. This covers an area of approximately 100 km^2^, which reflects a multicultural landscape with high potential for rural tourism and economic hub development. *A*_14_ is located in a strategic geographical location in Transylvania with great potential to generate internationally recognized trademark. The area has national and international level tourist attractions, fortified churches, well-known sustainable village models, natural reserves, a UNESCO heritage, etc. It used to be inhabited by Saxons hence generating both tangible and intangible and crucial cultural heritage. Therefore, this research demonstrates that the potential for rural development is high in rural areas that are located near locally, regionally, nationally or internationally recognized sites. Because we excluded from this study all urban areas, but also rural areas that are already touristic / economical developed, our results are significant, and can mark a starting point for in-depth research or future investment decisions.

In a context where the resources available for regional development are pressing decision-makers to maximize the effects of any development decision, the ranking of decision-making alternatives from the perspective of divergent criteria must provide the identification of the alternative that has the capacity to maximize the effects of resource allocation. In the case of resource allocation for tourism activities the direct effects related to the increase in the number of tourists for a given location has also an stimulating effect by creating an increase in the number of tourists and in adjacent areas, the so called concept of economic sharing benefits. From this point of view in a decision to allocate limited resources the decision-maker must direct those resources towards the alternative that will come closest to achieving the objectives. Decision makers often encounter with taking decisions on which area is prioritized to be developed within the limited budget. However, very few tools are available to determine appropriately development priorities for the diverse tourism objectives, perhaps because of a lack of systematized decision-making aids.

In our case, we put into question the prioritization of local development alternatives of 16 areas in the Transylvanian region in relation to the proposed criteria, criteria that shape the potential of each area to transform resources allocated to development into economic and social effects. The choice and allocation of development resources to an area, which are not condensed to convert to the maximum of these resources into benefits, will also have diminishing drive effect (stimulation) of the other areas in the immediate vicinity. Thus, directing resources to the area with the greatest potential will maximize the effects of development resource allocations and it creates a high drive effect for the adjacent area. Therefore, with the help of the AHP method, we aimed to determine the best alternative for rural development in the area of Southern Transylvania.

The obtained results can be used as a starting point in the development of rural development strategies applicable in Southern Transylvania. The analysis performed using the AHP method allowed the ranking of alternatives according to some criteria established following concrete discussions with local stakeholders. The most important findings can be further chosen for an in-depth analysis, in which the alternatives with the highest overall priorities can be analyzed based on an economic approach, thus extending the research.

Based on the results obtained in this study, we propose that stakeholders consider the investment decision for the development of a heritage business model in the area indicated by *A*_14_. The tourism development potential of *A*_14_ is proven by the scores obtained by applying the AHP method. Investments in this area will lead, in turn, to the development of neighboring areas, following the same assumed principle, that the existing trademark in the area contributes to the growth of tourism in neighboring areas. Our results can contribute to the enhance of regional and national strategies for rural development and resource allocation that may support heritage rural tourism and stimulate economic growth improving the quality of life in many of the less developed areas of Southern Transylvania.

## 6. Conclusion

The results outweigh the investment potential in rural areas with partially/remotely capitalized cultural heritage. The research also highlights the importance of existing TCEs for increasing competitiveness among cultural and/or natural heritage and tourism markets. Our research allows the recognition, ranking and selection of entrepreneurial development opportunities in rural areas with cultural heritage potential, with a positive impact on local economic and social sustainable development. Therefore, this study’s results show that cultural heritage, trademark, as well as the facilities offered by the analyzed areas, significantly contribute to choosing the best alternative for entrepreneurial development. Moreover, the criteria’s evaluation performed by the experts strengthened these results. Therefore, the level of accessibility of a place is not necessarily important for the investment decision. However, the history of the place, transposed in elements of cultural heritage, both tangible and intangible, is important. The best investment decision’s alternative (*A*_14_) is located near the national and European interests’ cultural heritage elements. The consistency ratios of the matrices (below the acceptable value of 0.10) confirm the accuracy of our results. The cultural tourism models developed in this area are innovative and integrate the area into the European cultural landscape.

As far as we know, our study is the first of this kind to examine Southern Transylvania and among the few studies in the cultural heritage capitalization sector. In addition, this paper contributes to the development of the specialized literature while few studies imply a similar research with three level decision-tree with a number of 16 alternatives.

The value of the research consists in the permanent communication held with the business environment and with the local authorities, precisely in order to identify the most relevant aspects for the development of a research with high applicability in the field. For data collection and criteria selection, qualitative surveys were conducted based on questionnaires addressed to local stakeholders in the selected localities from project eligible areas hence organizing several focus groups. The research results underlined the higher development potential for heritage goods and services to which new trademarks can be associated. Experts from five different but convergent professional backgrounds were selected to validate the needs for an innovative business model development (which includes cultural heritage local hub development) in rural areas. The study’s results can be used by public authorities as foundation for a sound strategic development of the area with economic potential from tangible and intangible heritage (re)interpretation for the benefits of everybody, according to Agenda 2030 goals implementation in rural areas.

In addition to the theoretical implications of this study, some practical implications are underlined. The results of our research highlight the tourism development potential of *A*_14_, which provides a generous area in which stakeholders can elaborate various heritage-based business models. Moreover, the examples of successful entrepreneurship models already existing in certain areas of Southern Transylvania can provide support in starting the investment process. Furthermore, with the help of the AHP method, taking into account the criteria established together with the local entrepreneurial environment, we narrowed the area of interest for the potential for tourism and entrepreneurial development in the area of Southern Transylvania. Thus, we propose that in the future the alternatives with the highest overall priorities should be subjected to in-depth economic analyzes.

MCDM, in general, is an analytical method that involves subjective decision makers. Even though we measured the consistency of the comparison matrices during the entire process of evaluation, the experts’ opinion in assessing weights and priorities may indicate subjectivity issues. Thus, we considered this a limitation to our research.

For further research, the following directions were identified: the extension of the monographic analysis and application of the AHP method for the second area selected in the ongoing Port Cultural project, for Bucovina in the Nord-East of Romania; the extension of the study by adding other two criteria that will allow on the one hand the transition to the development of smart villages that use digital technology to promote the local cultural heritage digitization the tourism product to capitalize the cultural heritage objective and promoting multisensory experiences in-situ [99] and on the other hand, the identification of interconnection possibilities at national / regional or European levels based on the common values of historical heritage (European cultural routes).

## Supporting information

S1 Text (DOCX)

S1 Appendix Defining the clusters that form the decision alternatives (https://osf.io/qwm9b/). (DOCX)

S2 Appendix Criteria description (https://osf.io/hdkng/). (DOCX)

S3 Appendix Criteria evaluation matrix (https://osf.io/t5jkm/). (DOCX)

S4 Appendix AHP data and methodology (https://osf.io/qdtze/). (XLSX)
