# Supplementary material for: Trademark potential increase and entrepreneurship rural development: A case study of Southern Transylvania, Romania
Source: PLoS One. 2021 Jan 15;16(1):e0245044. doi: 10.1371/journal.pone.0245044 (PMC7810323; doi:10.1371/journal.pone.0245044)
Supplement: S1 Text — (DOCX) [file pone.0245044.s001.docx]

Supporting information can be found at https://doi.org/10.17605/OSF.IO/BQPYK.
