# Supplementary material for: Trademark potential increase and entrepreneurship rural development: A case study of Southern Transylvania, Romania
Source: PLoS One. 2021 Jan 15;16(1):e0245044. doi: 10.1371/journal.pone.0245044 (PMC7810323; doi:10.1371/journal.pone.0245044)
Supplement: S1 Appendix — (DOCX) [file pone.0245044.s002.docx]

*S1 Appendix Defining the clusters that form the decision alternatives*

| **Crt. no.** | **Delimitation of the area** | **Elements of natural and anthropic heritage** | **Air quality / Air pollution elements** |
| --- | --- | --- | --- |
| 1 | Deda – Răstolița-Andreneasa- Lunca Bradului-Meștera-Stânceni  *Geographical location: Mureș Gorge, on both sides of the boundaries of the volcanic mountains Călimani and Gurghiu, distance from Tg Mureș: 50-80 km, European Road E578* | ***A. Potential Heritage for Trademark creation***  - the ethnographic museum from Deda that exposes traditions, objects and customs specific to the area of ​​the Mureș Gorge, dating back over 200 years;  - cultural route Via Transilvanica, Călimani route, being the longest segment in Romania that composes this cultural route (142 km). Objectives: Posmuș Mansion (1752).  - The Mureș Valley Festival (from Răstolița), with traditional specifics - gastronomy and folk art, held annually in July;  ***B. Other aspects that define the area***  - the largest population of Romanian brown bears in Romania (Gurghiului mountains);  - Călimani National Park (24566 ha) is home to rare species of animals and plants protected by the law (having several nature reserves registered in the Natura 2000 program, part of UNESCO's heritage); The Călimani-Gurghiu site is included in the Natura 2000 program (SPA type site) which include 13 habitats, of which 4 are of major international importance (species of birds, mammals, reptiles, fish and invertebrates);  - volcanic relief that houses rare land formations in Romania (vertically oriented basalt columns in Gurghiu mountains in Meștera) and in Europe (volcanic mill caves in Andreneasa: first ever discovered volcanic mill caves in the world), the mixed type nature reserve Lake Iezer in Călimani, etc.;  - mineral water springs from Călimani and Gurghiu Mountains;  - numerous mountain routes (around 30) with easy to medium level of difficulty, accessible for all the categories of tourists;  - hunting area (wild bears; horseback riding on Ilvei Valley, route of rides via Maria Therezia (through the highest Călimani Mountains peaks);  - accommodation possibilities in cottages, pensions, camping; | - air quality: *excellent*  No major pollutant factor is identified in the surrounding area. |
| 2 | Gornești - Gurghiu - Glăjărie - Idicel Pădure – Brâncovenești  *Geographical location: Târnavelor Plateau and Gurghiului Mountains, distance from Tg Mureș: 15-50 km, European Road E60, County Road* | ***A. Potential Heritage for Trademark creation***  - Teleki Castle (Gornești) - historical monument of national interest - dating from 1778, built in Baroque style, hosts the annual "Awake" electronic music festival in August.  - the castle from Brâncovenești (15th century), which belonged to several rulers of Transylvania; the village museum from Idicel-Pădure which presents features of the old houses of over 100 years.  - The Gurghiu Valley Festival in the village of Ibănești, held in August, has traditional specificity, in which the traditions, the dance and sing, the popular clothing specific to the area bring together the areas of the Gurghiului Valley;  - area famous for wild bear hunting, dating back to the Middle Ages; The Royal House of Romania participated in the hunting sessions, which built the hunting castle from Lăpușna (which is unfrequented today).  - Călimani cultural route of Via Transilvanica (Gornești, Reghin) during which you can visit the city of Reghin (the violin factory in Reghin, 90% of the activity is still done manually).  ***B. Other aspects that define the area***  - natural tourism in the reservation area of Gurghiu Mountains (camping, lodging in cottages, tourist routes, bear and lynx lynx - a rare species in Europe frequently found in the Romanian Carpathians - observers,)  - mountain routes to the highest peak of the Gurghiu mountains (Saca Mare peak 1680 m) starting from Gurghiu and other low or medium difficulty mountain paths (over 20 mountains routes). | - air quality: *very good*  - pollution source: Reghin chemistry works |
| 3 | Mădărași - Herghelia - Tăureni-Zau de Câmpie  *Geographical location: Transylvania Plain*  *distance from Tg Mures: 20-40 km, County Road* | ***A. Potential Heritage for Trademark creation***  - cultural heritage: the Ugron castle (1911) in Zau de Câmpie and the dendrological park around it; Banffy Mansion in Gheja (19th century), Sărmașu Museum of Religious Art;  - the lavender festival (Mădărași) in June;  - horse farm (Herghelia); Herghelia recovery center - vegetarian food from local products; bio certificated beekeeping farm, Herghelia agrotourism guesthouse;  - Reservation of steppe peonies (Zau de Câmpie), which bloom at the end of April. This botanical nature reserve, protected by national interest, is unique, being the only such reserve in Europe at an altitude of over 400 m.  ***B. Other aspects that define the area***  - leisure lakes (Tăureni - Zau de Câmpie), ideal for fishing activities;  - annually in April, a regional afforestation of the area is kept, accessible to volunteers. | - air quality: *very good*  - pollutant sources: „Ludus” Sugar Factory, „Azomures” Chemical plant |
| 4 | Recea - Ogra - Oarba de Mureș  *Geographical location: Târnavelor Plateau, Lunca Mureșului, distance from Tg Mureș 10-30 km, European Road E60* | ***A. Potential Heritage for Trademark creation***  - Recea Monastery, a heritage of national interest, which incorporates the only church in Europe (and the largest) made entirely of mosaic, built in 1991.  - Haller Castle, a historical monument of national interest, built in the 17th century by the Haller family in Baroque style, near the European road E60. Today it is an event center that functions as a pension - restaurant - wine cellar.  - historical monument from Oarba de Mureș, arranged in honor of the Romanian Heroes of the Second World War on a large surface, where over 3500 heroes were buried in the Oarba de Mureș Massacre, on September 17, 1944 (where over 11000 Romanian soldiers died, becoming the bloodiest fight conducted by the Romanian Royal Army on the Western Front).  ***B. Other aspects that define the area***  - high level of accessibility to road, train or air infrastructure (airport, highway and main railroad in this area);  - in the proximity of cultural and medical center of Târgu-Mureș | - air quality: *very good*  - pollutant emissions: „Azomures” Chemical plant |
| 5 | Vălișoara - Colțești - Râmeț - Gârbova de Sus - Gârbova de Jos  *Geographical location: Trascăului Mountains, Apuseni, distance from Alba Iulia 20-40 km, County Road* | ***A. Potential Heritage for Trademark creation***  - the ruins of the fortress of Trascău, dating from the 13th century (Colțești);  - Secuiesc Mansion from Colțești, built at the beginning of 2000, Hungarian restaurant and guesthouse;  - the Temple of the Knights Castle, over 700 years old, restored and transformed into a restaurant and boarding house in the Vălișoarei Gorges;  - numerous historical monuments specific to the area (churches, fortresses - Urieșilor Fortress Gârbova, Roman settlements discovered near Vălișoara, Colțești, Gârbova)  - Râmeț Monastery, located 18 km from the main European road, a historical monument of national interest, being one of the oldest monastic settlements in Transylvania (12th century);  ***B. Other aspects that define the area***  - natural tourism through the Trascăului mountains: Valisoarei Gorges (Aiudului), Râmețului Gorges, paleonthological reserve "Pârâul Popii" from Gârbova de Sus;  - leisure activities: paragliding flight on the Trascăului Mountain Peaks, bike rides, field engines, mountaineering, caving, etc. | - air quality: *excellent*  -pollutant: chicken farm and  slaughterhouse ”Avicola” |
| 6 | Galda de Jos - Cricău - Craiva - Bucerdea Vinoasa - Ampoița - Sub Piatră  *Geographical location: Trascăului Mountains, Apuseni, distance from Alba Iulia 10-40 km, County Road* | ***A. Potential Heritage for Trademark creation***  - Kemeny Castle, a historical monument dating from the 17th century, the Church of the Birth of Mary (1715),  - areas of historical interest, where Roman and Saxon settlements were discovered, archaeological sites of national importance - Ampoița, Cricău, Galda de Jos, Galda de Sus;  - the national folklore festival „Sus, sus, sus, la moți, la munte" Câmpeni (organized in July), the Rural Tourism Fair in Albac (organized in late summer)  - the Dacian fortress Apoulon, located on the Piatra Craivii peak (1078 m), in Craiva commune, dating from the 1st century b. Chr.  - Ampoiței Keys, Huda lui Papură Cave (the longest cave in Romania, with the highest entrance mouth, the longest watercourse, etc.), numerous nature reserves (wrenches, caves, waterfalls), historical monuments (Saint Paraschiva Monastery XVIII century)  - rich history of the area, there is evidence that this area has been permanently inhabited for the last 5000 years (Dacians and Romans)  ***B. Other aspects that define the area***  - natural wealth, including: wineculture, forests, but also gold. Nearby (Baia de Arieș) the gold was exploited by the Saxons; Gălzii Gorge, Bulzul Gălzii nature reserve, numerous orchards (Galda de Jos).  - Rafting activities, paragliding, tourist routes to karst formations of national interest - in the area or in the vicinity are among the most spectacular limestone formations in Romania: caves, wharves, gorges. | - air quality: *excellent*  No major pollutant factor is identified in the surrounding area. |
| 7 | Vințu de Jos - Căpâlna - Daia Română - Berghin – Ciugud  *Geographical location: Secașelor Plateau, Târnavelor Plateau, distance from Alba Iulia 10-40 km, European Road E81, European Road E68, Highway A1, National Road DN67C, County Roads DJ106, DJ107* | ***A. Potential Heritage for Trademark creation***  - numerous historical monuments of national interest (Vințu de Jos: Martinuzzi castle, 1551, one of the representative buildings of the Transylvanian Renaissance, initially a Dominican monastery - the only one of its kind in rural Transylvania; a fortified evangelical church from the 14th to the 19th centuries; Daia Română: the Greek Catholic church "Saint Trinity", painted in the eighteenth century, dated around 1664-1666; Berghin: the wooden church of St. Peter, built in 1707, relocated to Berghin in 1900 from Gârbova de Sus; .)  - historical area of ​​national importance, where Dacian settlements were discovered (Dacian fortress from Căpâlna - included in the list of UNESCO World Cultural Heritage - 2nd century b. Chr. - 106 p. Chr., archaeological site Berghin, necropolis, archaeological site Ciugud , Neolithic and early medieval settlements Ciugud, Neolithic settlement Daia Română)  - Festival of traditions and customs "Culture for culture", Wooden Day, etc.  - cultural route Via Transilvanica, the Valea Mureșului route (Geoagiu - Alba Iulia - Blaj, the Vințului corridor - the Mureș Valley course on the Aiudului corridor)  ***B. Other aspects that define the area***  - area with salt springs (Daia Română), natural and geological reserves (Râpa Roșie);  - in proximity to Alba Iulia city, which houses the biggest Vauban citadele in Europe. | - air quality: *excellent*  No major pollutant factor is identified in the surrounding area. |
| 8 | Jidvei - Balta Fortress - Bazna - Agârbiciu - -Șeica Mare - Metiș - Bârghiș - Moșna – Cund  *Geographical location: Secașelor Plateau, Târnavelor Plateau, Hârtibaci Plateau, distance from Sibiu 20-50 km, National Road DN14, County Roads DJ107, DJ141, DJ 142* | ***A. Potential Heritage for Trademark creation***  - historical monuments of national interest (archeological site Cetatea de Bală; foundations, fortifications with earth wave of the old Fortress of Balta, castro, Jidvei, Șeica Mare: evangelical-Lutheran church, built in 1300, which marks the transition from romantic to gothic style).  - fortified churches (Cetatea de Baltă, XIV century, Agârbiciu, XV century; Bazna, XIV century; ensemble of evangelical churches Jidvei, Bazna, Moșna)  - Bazna rural complex, Tobias castle, baroque style, XVIII century, the Castle of the Bolyai - Șeica Mare family, donated by Sigismund Bathory to Mihai Viteazul in 1598;  - spa resort of national interest Bazna - mineral springs and salt springs, the monument of the Romanian heroes of the Second World War Bazna.  - cultural route Via Transilvanica, route Terra Saxonia (Blaj-Mediaș-Moșna), where you can visit tourist attractions from the localities you cross)  - events: The National Folklore Festival "Golden Grapes" Jidvei (organized in September every year), the days of the open gates Cetatea de Baltă (in June), etc.  ***B. Other aspects that define the area***  - numerous accommodation possibilities in traditional houses, preserving the traditions and architectural elements specific to the Hungarian and Saxon culture in the area: Jidvei, Valea Verde Resort, Bârghiș  - touristic cultural routes addressed to all kind of tourists | - air quality: *very good*  - pollutant emissions: car emissions |
| 9 | Cristian - Cisnădioara - Gura Râului - Păltiniș - Prislop - Boița – Tălmăcel  *Geographical location: Târnavelor Plateau, Hârtibaci Plateau, Parangului Mountains, distance from Sibiu 20-50 km, National Road DN7, County Road DJ106* | ***A. Potential Heritage for Trademark creation***  - historical monuments of national interest: archaeological sites, civil constructions Boița; fortified churches (Cisnădioara, XIII century; Boița, XIV century; Tălmăcel), of local interest: wooden bridge, wooden houses, hydraulic textile assembly Gura Râului; Turnu Rosu castle from Boița (15th century), the oldest functioning tube organ in Romania (Cisnădioara).  - Cisnădioara cultural events center (shows, concerts, exhibitions);  - „Festivalul Brânzei și al Țuicii” from Rășinari, Sibiu, with specific traditionally culinary preparations from the region, organized at the end of August - beginning of September).  - the Apsara trance music festival from Ludoș (organized in August), Sibiu; The festival "Up on the mountain in Jina from Mărginimea Sibiului (organized in the middle of summer), one of the oldest - festivals in the county, which aims to preserve and enhance the traditions and customs, songs and games of the jinaries and their way of life.  ***B. Other aspects that define the area***  - developed bicycle touring (Cisnădioara - Păltiniș), mountain trails for beginners and advanced;  - area of ​​tourist interest: museums (Museum of Saxon traditions in Cisnădioara), Păltiniș resort - winter sports, dam of the Gura Râului accumulation lake, etc. | - air quality: *very good*  -pollutant: Mârșa mechanical plant |
| 10 | Porumbacu de Sus- Porumbacu de Jos - Cârțișoara - Hosman - New Romanian - Saturday - Cincu - Cincșor - Dealu Frumos  *Geographical location: Târnavelor Plateau, Hârtibaci Plateau, Parângului Mountains, Făgăraș Mountains, distance from Sibiu 40-60 km, distance from Brașov 40-60 km, National Road DN7C, European Road E68, County Road DJ104* | ***A. Potential Heritage for Trademark creation***  - The ethnographic and memorial museum "Badea Cârțan" from Cârțișoara; Brâncoveanu Castle from Sâmbăta de Sus, Brâncoveanu Sâmbăta Monastery; mountain trails, karst and mountainous reliefs; The school from Cincșor (with possibility of accommodation within its premises).  - festivals: Holzstock music from Hosman; the festival of culture and arts in Nocrich; horror film festival "Full Moon" from Biertan, (organized at the end of August);  - the Sâmbăta X3 festival, with sporting activities, concerts and culinary experiences (Sâmbăta de Sus, at the end of July)  - Historical monuments: the fortified church Cincșor (13th century), Dealu Frumos (13th century), the whole of Brukenthal castle in Sâmbăta de Jos (1750-1760, owned by the family of the governor of Transylvania of that period, on land offered for rent by Empress Maria Thereza;  ***B. Other aspects that define the area***  - Cincu US military base, which attracts hundreds of curious visitors every year, although this military base is not open to the general public.  - numerous tourist attractions: the Castle of Lut "Valea Zânelor" Porumbacu de Sus, restaurant and hotel with a panorama over the Făgăraș Mountains, built in 2014, being part of a story landscape; Sâmbăta climatic resort; agrotourism pensions, National Park Făgăraș Mountains Reservation, trout, Lake Tatar, Transfăgărășan road, which ranks second in altitude in the classification of alpine roads in Romania, being proposed by the makers of Top Gear as the most spectacular road in the world.  - mountain routes, which include the faimous route to Moldoveanu Peak (2544 m), the highest peak in Romanian Carpathians (medium level of difficulty)  - the geographical center of Romania from Dealu Frumos, Sibiu county. | - air quality: very good  -pollutant emissions: car emissions |
| 11 | Șinca Veche - Poiana Mărului - Măgura - Cave - Fundățica – Feldioara  *Geographical location: Hârtibaciului Plateau, Piatra Craiului Mountains, Bucegi Mountains National Road DN73, County Road DJ 112, National Road DN13* | ***A. Potential Heritage for Trademark creation***  - historical monuments: Dacian settlements (Șinca Veche, Feldioara, Rotbav) the rock monastery (Șinca Veche, seventeenth century), the church assembly "Birth of St. John the Baptist" (Șinca Veche); archaeological site Feldioara, civil settlement, Roman castle Feldioara, the assembly of the evangelical church Feldioara, the Feldioara Fortress (fortified Gothic fortress, built in the 13th century, inscribed in the list of historical monuments of national interest), the monument of the 16 Saxon students fallen in battle; Râșnov Fortress (fortified ensemble dating from the 14th century, located at the top of a limestone hill, being one of the best preserved fortified assemblages in Transylvania).  - in the area: annual rock music festival (Râșnov) organized annually in July;  - the Rucăr-Bran corridor which houses many tourist attractions consisting of built heritage: Bran castle (the castle mentioned by Bram Stocker in Dracula novel), Peleș castle of the royal family, etc.  ***B. Other aspects that define the area***  - agrotourism pensions, rural tourism, natural tourism (mountain and tourist routes to mountain peaks, caves, gorges and gorges, speleology, hiking etc. - over 30 routes alternatives with many levels of difficulties, accessible for all kinds of tourists), cyclotourism in the Piatra Craiului Mountains Nature Reserve, accommodation in pensions or traditional Romanian houses, the possibility of tasting local gastronomy. | - air quality: *excellent*  No major pollutant factor is identified in the surrounding area. |
| 12 | Zizin - Teliu - Prejmer - Ozun – Chichiș  *Geographic location: Târnavelor Plateau, Hârtibaci Plateau, Parângului Mountains, Făgăraș Mountains, distance from Sibiu 40-60 km, distance from Brașov 40-60 km, County Road DJ 103, National Roads DN10, DN11* | ***A. Potential Heritage for Trademark creation***  - historical monuments: fortified churches (Chichiș, Prejmer, Ozun, 14th century); Chichiș archeological site, Dacian fortification, Prejmer Unitarian church - listed in the UNESCO World Cultural Heritage list, Chichiș wood church (1740), Prejmer rural site, Prejmer evangelical confessional school (1848), Roman settlement Măgheruș, Denes mansion (Szekler style, dating from the 1840s), the rural complex "Wood houses" Chichiș, etc.  - events: Visit Covasna Marathon (organized in September), which incorporates a cultural event  ***B. Other aspects that define the area***  - leisure areas (Nature Park Chichiș, Lake Chichiș), Hanul Gulyas Chichiș restaurant with traditional Szekler specificity;  - natural reserves: the forest and the eutrophic marshes Prejmer, the birch from Reci and the ponds from Ozun-Sântionluca; mineral water springs. | - air quality: *very good*  -pollutant emissions: the peripheral industry in Brașov and Săcele |
| 13 | Racoș - Cața - Ocland - Vârghiș - Tălișoara - Biborțeni - Bățani - Malnaș-Băi - Bixad - Băile Balvanyos – Tușnad  *Geographical location: Harghita Mountains, Baraolt Mountains, Hoghiz Depression, Boboc Mountains, County Roads DJ 131, DJ 132, DJ 122, European Road E578* | ***A. Potential Heritage for Trademark creation***  - historical monuments: archaeological site Racoș, Dacian fortification Racoș; archaeological site Malnaș-Băi, Geto-Dacian settlement Bixad, Bixad Fortification, Rika Fortress from Racoșul de Sus, fortified church from Cața (13th century), Kustaly Ocland fortress (9th-12th century), Ocland archeological site, ruins of the fortress Tușnad (12th century), Daniel Castle from Tălișoara (1620-1680), with architectural elements specific to the Szekler, Large Mansion - the house of the writer Benedek Elek (1906), etc.  - the festival "Day of the Oak" from Homorod commune (sporting activities, local culinary experiences), organized in October; the "Day of the Stone" festival in Racoș, organized in November, "Haferland Week" which aims to promote the Saxon culture of Brașov County (bicycle activities, culinary experiences, crafts workshops) since July; the balcony from Tusnad village, organized in the summer months; Covasnene Equestrian Days, Olteni, held at the end of June;  ***B. Other aspects that define the area***  - Gastro Lab restaurant with gastronomic specificity of the area, Turia;  - numerous natural tourist attractions: Vârghișului Gorges, mineral water springs, Sf Ana Lake (the only volcanic lake in Romania), Balvanyos Bath, Cetăţii Peak (Natura 2000 site), extinct volcano from Racoș, basalt quarry from Racoș, the emerald lake from Racoș (the only natural lake of accumulation with rainwater) vertical basalt columns - the highest in Romania; paleontological reserve in Racoș - the only paleontological reserve in Romania that harbors species of ammonites. Easy access for visitors to all tourist attractions | - air quality: *very good*  *-* pollutant emissions: Hoghiz cement company |
| 14 | Criț - Saschiz - Hunters - Prod – Sângeorgiu de Pădure - Bezid - Praid - Corund – Zetea  *Geographic location: Târnavelor Plateau, Hârtibaciului Plateau, Praid Depression, Harghita Mountains, European Road E60, National Roads DN14, DN13A, County Road DJ 151.* | ***A. Potential Heritage for Trademark creation***  - historical monuments: Firtos fortress in Corund (2nd century), Romanian guard tower in Ocna de Sus (4th century), Rapsone Praid fortress (11th-12th centuries), Geto-Dacian fortress Sub Fortress (1st century) , unpaved stone wall, earth wave, defense ditch Sub Cetate, the ruins of the Saschiz fortress (13th century), the Museum of Straw Hats (hosted by an old house over 150 years old), etc.  - numerous tourist attractions: Praid salt lake, salt canyon from Praid, Lake Bezid, Zetea dam, Zetea wild animal reserve, the geological reserve Dealul Melcilor (Corund), mineral and salt water springs, the Corund Village Museum, the ceramic pots festival in Corund (August), the daffodils festival in Dealu (May), the Szekler equestrian holiday Gheorgheni (August).  - annual meeting of international bicycle passionate tourists, held during a week in the area of Saschiz in the middle of the summer  ***B. Other aspects that define the area***  - numerous leisure areas: the horse farm Prod; Bezid accommodation, pottery workshops and Corund handicraft products exhibition; organized tourist routes in the Zetea area (with cart, observation of the Carpathian bears in the natural habitat, creative workshops, secrets of the roasting of the Szekler col)  -accommodation possibilities, bicycle touring (internationally recognized bicycle-routes), agrotourism pensions (Zetea, Sub Cetate), mountain tours in Harghita county through the e-bike service; horse-drawn sleigh ride (in winter); ski slopes, sport fishing (Zetea, Sub Cetate, Târnava Mare), horse riding center (Izvoarele), paragliding Hășmaș Mountains, Târnava Mare rafting, "Balu" park adventure, mountain tours with montaincart, atv, segway, snowmobiles, the dog sled, the tourist route of the Saxon fortified churches in the Rupea area. | - air quality: *very good*  Pollutant source: car emissions |
| 15 | Siculeni - Frumoasa - Lunca de Jos - Sândominic - Izvoru Mureșului  *Geographical location: Hășmașu Mare Mountains, Ciuc Depression, Ciuc Mountains distance from Miercurea Ciuc 20-50 km, European Road E578, National Road DN12A* | ***A. Potential Heritage for Trademark creation***  - historical monuments: Ciceu village citadel (13th century), the whole Roman-Catholic church "Sf. Nicolae ”Frumoasa (14th century), the ensemble of the Roman Catholic church“ Sf. Dominic "(13th century) from Sandominic, the Siculicidium column - historical monument in memory of the Siculeni massacre  -Near the natural reserve from Reci, the Dacian fortress Covasna, which attests the presence of the Dacians and Romanians in the present territory of Covasna;  ***B. Other aspects that define the area***  - numerous tourist attractions: Frumoasa lake, Caracau railway viaduct, mountain trails in Ciucului and Hășmașu Mare mountains, Mureș tourist spring, mineral water springs, Mureș spring recreational resort, Olt spring etc.;  - accommodation possibilities, tourist routes of easy to medium difficulty. | - air quality: *excellent*  No major pollutant factor is identified in the surrounding area. |
| 16 | Red Lake - Lăzarea - Ditrău - Secu - Bilbor - Corbu – Tulgheș  *Geographical location: Hășmașu Mare Mountains, Giurgeului Depression, Bilbor Depression, Ceahlău Mountains, distance from Miercurea Ciuc 40-80 km, National Road DN 12C, European Road E578, National Road DN15.* | ***A. Potential Heritage for Trademark creation***  - historical monuments: archaeological site Lăzarea, settlement medieval era Lăzarea, the ensemble of the Roman Catholic church Bilbor (1895), etc.  - tourist attractions: Red Lake - the only natural dam lake in Romania, the Ditrău church (built in the early 1900s in neo-gothic style), the Ditrău evangelical church (historical monument of local and national interest, dated from 1546-1557), the hero's monument from Secu, built in memory of 771 Romanian soldiers who lost their lives in this place during World War I, the mineral waters of Bilbor (known for its high levels of sulfur and nitrates), the mineral water springs from Borsec (with different properties, recognized for the benefits in the treatment of some diseases), Tulgheș village, where there are different buildings and strategic places used in World War II);  - events: the Carpathian Mountains Festival, Tulgheș (end of July), with popular specific, which promotes the folk arts specific to the area, Borsec Days (end of July, beginning of August), the folk art festival Durău (beginning of August)  ***B. Other aspects that define the area***  - accommodation possibilities, tourist routes in the area or in the vicinity (Borsec resort, Durău resort, Ceahlău Natural Park, Bicaz Gorges, etc.). Area with developed ecotourism (recreational, tourist, cultural activities, hang gliding, ascents, viewing points, etc.).  - In the vicinity there is the Ceahlau Natural Park, which houses gorges, waterfalls, high and spectacular peaks, very visited by tourists. | - air quality: *very good*  - pollutant emissions: Heidelberg cement company, Romania |

Source: authors’ own research
