# Supplementary material for: Trademark potential increase and entrepreneurship rural development: A case study of Southern Transylvania, Romania
Source: PLoS One. 2021 Jan 15;16(1):e0245044. doi: 10.1371/journal.pone.0245044 (PMC7810323; doi:10.1371/journal.pone.0245044)
Supplement: S2 Appendix — (DOCX) [file pone.0245044.s003.docx]

***S2 Appendix Criteria description***

| **Criteria*** | **Sub-criteria**** | **Description** |
| --- | --- | --- |
| Trademark | Age | Age was evaluated considering the addressability to two categories of potential tourists: young and mature.  Categories of interests considered in are the presence of cultural and natural objectives and different entertainment events that can be use or developed as trademark.  Facilitated access to interests, multilanguage support and local involvement in creating and accessing authentic experiences (e.g. guiding services, local transport facilities like old carriages, old trains etc).  Authentic experiences are evaluated in accordance to the value of local traditions and the capacity to offer interactions with local culture (food, heritage, nature etc.) |
|  | Interests |  |
|  | Infrastructure |  |
|  | Authentic Experiences |  |
| Accessibility | Social Media Presence | The criterion "accessibility" was evaluated taking into account the access to road, rail, telephone, etc ; by the visibility of the places through social networks, but also on sites promoting the tourist offer (Booking, TripAdvisor, etc.). |
|  | Access |  |
|  | Remote |  |
| Landscape | Air Quality | The criterion "Landscape" was evaluated by the existence of natural reserves (considering uniqueness, beauty, type of reservation, etc.) and by the possibility of carrying out tourist routes (pedestrian, bicycle paths, shelf, etc.) in the area or in the surrounding area. The air quality index reported in the area was analyzed, considering the database and the annual reports of the National Agency for Environmental Protection. |
|  | Natural Reservations |  |
|  | Natural Tourist Routes |  |
| Cultural Heritage | - | The criterion "Cultural Heritage" was evaluated from the perspective of the existence of the cultural heritage objectives, their value, the category and the attractiveness for certain categories of tourists. |
| Facilities | Accommodation | Presence of accommodation facilities, easy to find and access, quality addressability to a large panel of tourists.  Number and quality of restaurants and food access facilities  Presence of organized and potential tourist activities  All have been evaluated according to the presence on tourist source of information, distance to the center of the evaluated areas and tourist reviews. |
|  | Restaurants |  |
|  | Tourist Activities |  |

*Note:*

** The criteria were chosen by the researchers based on the results of the focus groups and in-depth interviews.*

*** The sub-criteria were established by the experts who evaluated the main criteria, using professional reasoning.*

Source: authors’ research
