# Supplementary material for: Trademark potential increase and entrepreneurship rural development: A case study of Southern Transylvania, Romania
Source: PLoS One. 2021 Jan 15;16(1):e0245044. doi: 10.1371/journal.pone.0245044 (PMC7810323; doi:10.1371/journal.pone.0245044)
Supplement: S3 Appendix — (DOCX) [file pone.0245044.s004.docx]

***S3 Appendix Criteria evaluation matrix***

| *The weight assigned to each criterion* | | | | | | | |
| --- | --- | --- | --- | --- | --- | --- | --- |
| Criteria | | Trademark | | Accessibility | Landscape | Cultural Heritage | Facilities |
| Trademark | | 1.000 | | 4.000 | 3.000 | 2.000 | 1.000 |
| Accessibility | | 0.250 | | 1.000 | 0.333 | 0.2000 | 0.250 |
| Landscape | | 0.333 | | 3.000 | 1.000 | 0.5000 | 0.500 |
| Cultural Heritage | | 0.500 | | 5.000 | 2.000 | 1.000 | 0.500 |
| Facilities | | 1.000 | | 4.000 | 2.000 | 2.000 | 1.000 |
| *Normalized matrix* | | | | | | | |
| Criteria | | Trademark | | Accessibility | Landscape | Cultural Heritage | Facilities |
| Trademark | | 0.324 | | 0.235 | 0.360 | 0.351 | 0.308 |
| Accessibility | | 0.081 | | 0.059 | 0.040 | 0.035 | 0.077 |
| Landscape | | 0.108 | | 0.176 | 0.120 | 0.088 | 0.154 |
| Cultural Heritage | | 0.162 | | 0.294 | 0.240 | 0.175 | 0.154 |
| Facilities | | 0.324 | | 0.235 | 0.240 | 0.351 | 0.308 |
| *Overall priorities* | | | | | | | |
|  | **0.3156** | | **0.0584** | | **0.1292** | **0.2051** | **0.2916** |

Source: authors’ own calculation
